# Detection of Single Nucleotide Polymorphisms by Fluorescence Embedded Dye SYBR Green I Based on Graphene Oxide

**DOI:** 10.3389/fchem.2021.631959

**Published:** 2021-03-31

**Authors:** Jiaoyun Xia, Tong Xu, Jing Qing, Lihua Wang, Junlong Tang

**Affiliations:** ^1^ School of Chemistry and Food Engineering, Changsha University of Science and Technology, Changsha, China; ^2^ Shanghai Institute of Applied Physics, Chinese Academy of Sciences, Shanghai, China; ^3^ School of Physics and Electronic Science, Changsha University of Science and Technology, Changsha, China

**Keywords:** graphene oxide, single nucleotide polymorphism, SYBR Green I, DNA sensor, fluorescence

## Abstract

The detection of single nucleotide polymorphisms (SNPs) is of great significance in the early diagnosis of diseases and the rational use of drugs. Thus, a novel biosensor based on the quenching effect of fluorescence-embedded SYBR Green I (SG) dye and graphene oxide (GO) was introduced in this study. The probe DNA forms a double helix structure with perfectly complementary DNA (pcDNA) and 15 single-base mismatch DNA (smDNA) respectively. SG is highly intercalated with perfectly complementary dsDNA (pc-dsDNA) and exhibits strong fluorescence emission. Single-base mismatch dsDNA (SNPs) has a loose double-stranded structure and exhibits poor SG intercalation and low fluorescence sensing. At this time, the sensor still showed poor SNP discrimination. GO has a strong effect on single-stranded DNA (ssDNA), which can reduce the fluorescence response of probe DNA and eliminate background interference. And competitively combined with ssDNA in SNPs, quenching the fluorescence of SG/SNP, while the fluorescence value of pc-dsDNA was retained, increasing the signal-to-noise ratio. At this time, the sensor has obtained excellent SNP resolution. Different SNPs detect different intensities of fluorescence in the near-infrared region to evaluate the sensor's identification of SNPs. The experimental parameters such as incubation time, incubation temperature and salt concentration were optimized. Under optimal conditions, 1 nM DNA with 0–10 nM linear range and differentiate 5% SNP were achieved. The detection method does not require labeling, is low cost, simple in operation, exhibits high SNP discrimination and can be distinguished by SNP at room temperature.

## Introduction

Single nucleotide polymorphisms (SNPs) are extremely important genetic variations in human genes (Martin et al., 2020). SNPs research is closely related to the development of pharmacogenomics and disease diagnosis, and is an important step toward the application of human genome projects (Li et al., 2019). At present, there are many methods for detecting SNPs (Uppu et al., 2016; Zhao et al., 2018; Prezza et al., 2019), including gene sequencing technology (Kim et al., 2016), capillary electrophoresis technology (Zhao et al., 2019), reversed-phase high performance liquid chromatography (HPLC) detection (Jones et al., 1999; Tonosaki et al., 2013), gene chip technology (Tu et al., 2018), etc. Although these methods can effectively detect SNPs, most of them PCR technology is required to amplify DNA (Qing et al., 2019), which is expensive and time-consuming. Furthermore, the probability of obtaining a false positive is high and the process is complex (Matsuda, 2017). Thus, a fast, economical and reliable SG embedded sensor is introduced to detect SNP. Such as, inserting SG into DNA double strands, and then immobilizing DNA on the surface of molybdenum disulfide-gold nanoparticles (MoS_2_@AuNPs), and MoS_2_@AuNPs quenches the fluorescence of SG inserted into the DNA double strands. So as to clearly distinguish the SNPs in the DNA sequence. (Yan et al., 2019). Using SG as an electrochemical indicator, SNP detection is carried out according to the strength of the SG intercalation between chains and the strength of the electrocatalytic reaction. SNPs show higher SG intercalation rate and lower current response. (Vasconcelos et al., 2018). But only with the participation of SG, it is difficult for the sensor to achieve high resolution and high accuracy SNP detection.

SYBR Green I (SG) is a dye with green excitation wavelength that binds to all dsDNA double helix minor groove regions, and has a very high affinity with dsDNA (Zhou et al., 2019). In the free state, SG emits weak fluorescence, but once combined with double-stranded DNA, the fluorescence is greatly enhanced (Dragan et al., 2012; Yi et al., 2019). Due to this unique property, SG has been widely used in electrophoresis, real-time PCR and other forms of DNA qualitative and quantitative analysis (Morozov et al., 2020; Wang et al., 2020). However, SG cannot completely distinguish between stable Watson-Crick structure and unstable structure formed by mismatch, so SG is rarely used for SNP analysis alone (Maruyama et al., 2003). In recent years, nano-quenchers have been developed in biosensing. Graphene oxide (GO) has a large surface-to-volume ratio and contains hydrophobic hexagonal rings that can interact with biomolecules (Chen et al., 2017; Mousavi et al., 2019). It is a good biosensor fluorescence quenching material. SNPs with loose double-stranded structures have stronger adsorption on the surface of graphene oxide through hydrophobic interactions between the bases of DNA and the hydrophobic surface of graphene oxide than fully complementary double-stranded DNA (pc-dsDNA) (Tikum et al., 2018). GO can specifically act on SNP and quench the fluorescence of the SG/SNP system. (Campbell et al., 2019). The GO-assisted SYBR Green I assay produced a large difference in fluorescence intensity between fully complementary DNA and base mismatched DNA. This improves the detection sensitivity and efficiency of fully complementary DNA and base mismatched DNA.

In this study, a novel biosensor based on the quenching effect of fluorescence-embedded SYBR Green I (SG) dye (Hur et al., 2019) and graphene oxide (GO) (Li et al., 2013; Wan et al., 2017; Jiang et al., 2020) was constructed. SG was inserted into the completely complementary DNA and base mismatched DNA respectively. The completely complementary DNA showed strong fluorescence, and then GO was added to enlarge the difference in fluorescence signal between pc-dsDNA and SNPs. According to the specific changes of the fluorescence signal, a clear SNP distinction is achieved. It provides a simple, fast and accurate strategy to solve the problem of SNP detection.

## Experimental

### Reagents and Apparatus

DNA oligomers were purchased from Shanghai Biotech Biotechnology Co. (Shanghai China). All of the DNA oligomers were purified by high performance liquid chromatography (HPLC). These sequences are shown in Supplementary Table S1 and were prepared with 100 μM by UV3010 (Hitachi, Japan) ready for use. Graphene oxide (GO) was provided by laboratory of physical biology, Shanghai Institute of Applied Physics, Chinese Academy of Sciences. All other reagents were of analytical reagent grade and used directly without treatment. Milli-Q water (18.2 MΩ) was used throughout the experiments. SYBR Green I (SG) was made up with DMSO as 10,000 × stock solution. The 10 mM phosphate buffer solution with 7.4 was prepared by mixing the stock solution of K_2_HPO_4_, NaCl, and KH_2_PO_4_ (10 mM PB, 50 mM NaCl, pH 7.4).

Fluorescence emission spectra were performed using a F-4500 fluorescence spectrophotometer (Hitachi, Japan) with a scan rate at 1,200 nm/min. The slits for excitation and emission were set at 5 nm/5 nm.

### Experimental Method

In a typical experiment, 10 μl of probe DNA (10 μM) and 10 μl of target DNA (10 μM) were hybridized in 20 μl of 10 mM phosphate buffer (20 mM NaCl, pH 7.4, PBS) for 30 min at room temperature. The mixture of 4 μl hybridization solution and 10 μl SG (×10) was placed in a fluorescence colorimetric plate, and then 986 μl water was added. The resultant solution was then added 5 μl of graphene solution (500 μg/ml) and was carried out immediately after mixing to ensure that the detection time was consistent after each addition of graphene solution, generally 15 s. During the detection, the excitation wavelength was 497 nm, and the scanning range was 507–650 nm. Each sample was measured five times.

## Results and Discussion

### Principle of Sensing SNP

**SCHEME 1:**
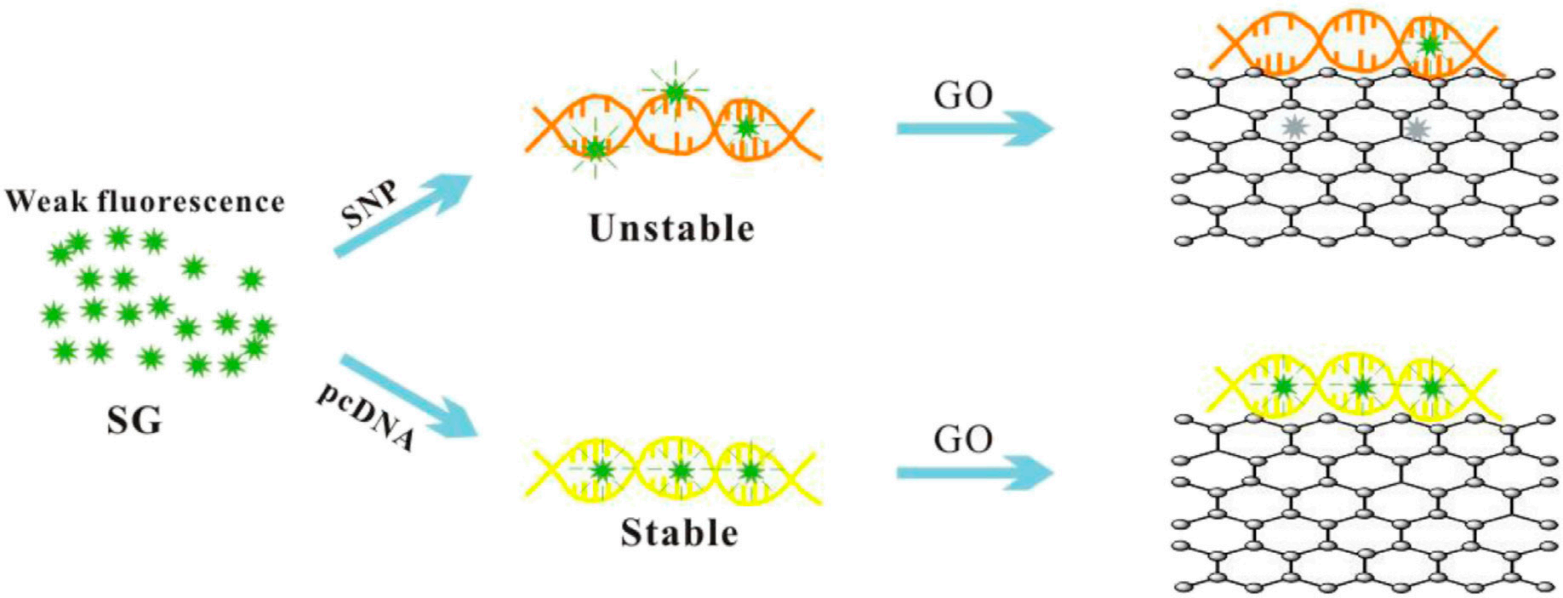
SNP assay sensor composed of graphene oxide (GO) and SYBR Green I (SG)

The experimental principle of the biosensor is illustrated in Scheme 1. SG is well embedded in fully complementary dsDNA and produces high fluorescence. However, a single matching sequence usually results in unstable DNA’s double helix, which exhibits poor SG embedding and low fluorescence (Zipper et al., 2004; Li et al., 2013). Graphene oxide enhances the unwinding of unstable SNP through strong GO/ssDNA interactions, thereby quenching the fluorescence from free SG and SG/SNP. The result is a high signal-to-noise ratio (S/N) and excellent SNP discrimination.

### Biosensor Identification SNP Based on GO Embedded SG

**FIGURE 1:**
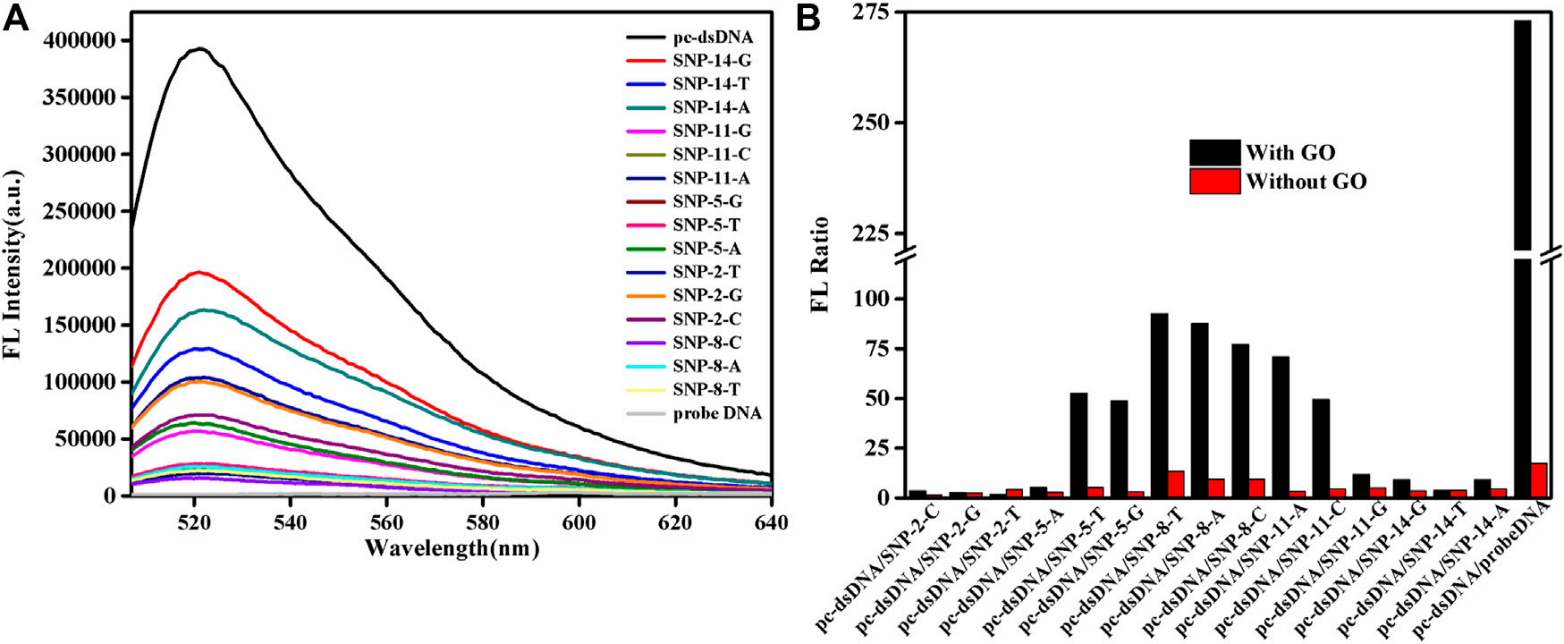
Emission spectra of a biosensor consisting of graphene oxide (GO) and SYBR Green I (SG) that detects single nucleotide polymorphisms (SNPs). **(A)** FL intensities of the samples after adding GO. **(B)** FL intensities as a function of various DNA/SG/GO or DNA/SG. The final concentration: [SNPs] = 10 nM, [probe DNA] = 10 nM, [pcDNA] = 10 nM, [GO] = 2.5 μg/ml, 10 mM phosphate buffer ( 20 mM NaCl, pH 7.4).

SG specifically embeds dsDNA, and the fluorescence value of SG/dsDNA is much higher than that of SG alone. Hence, SG is widely used in gel electrophoresis and PCR technology. This study examined 15-base SNPs, and base mismatches occurred every two bases, as shown in Supplementary Table S1. In Figure 1, the fluorescence value of the SG/pc-dsDNA group is higher than that of the SNP group containing mismatched bases. This shows that the structure of DNA containing mismatched bases is loose and the amount of SG embedded is small, so the fluorescence value is low. Supplementary Figure S1 is the fluorescence spectrum of the sample without GO, which shows that SG can distinguish between the pc-dsDNA and SNP groups. However, due to the low sensitivity and weak specificity of SG itself, the discrimination effect of pc-dsDNA and SNPs is poor. The fluorescence value of the SNP group is still high; particularly the base mismatch sequences at positions 2 and 14 have higher fluorescence values. It is evident that SG can specifically select SNPs. Because the effect was not ideal, GO was added to optimize the experimental results. Figure 1A is the fluorescence spectrum of the sample after adding GO. It is evident that the fluorescence value of pc-dsDNA is still high, but the fluorescence value of the SNP groups and probe DNA is extremely low. At this time, the background interference can be almost ignored, and the signal-to-noise ratio has also been greatly improved. Figure 1B visually lists the results of sample detection before and after adding GO. The ordinate is the fluorescence ratio of pc-dsDNA to SNP or probe DNA. The red band is the result of the experiment before the addition of GO. The fluorescence value of pc-dsDNA is about 5–15 times that of the SNP group and 20 times that of probe DNA. Its signal to noise is relatively low. The black band is the result of the experiment after adding 2.5 μg/ml GO. The fluorescence value of pc-dsDNA is about 20–120 times that of the SNP group and about 260 times that of probe DNA. The signal-to-noise ratio was significantly higher than that of the experimental group without GO, and the signal-to-noise ratio was significantly different with the base mismatch positions. The highest signal-to-noise ratio was at the base 8th position, and the lowest signal-to-noise ratio was at the base 2nd and 14th positions. This may indicate that GO can quench the fluorescence, reduce the background signal, improve the signal-to-noise ratio, and improve the sensitivity and efficiency of SNP detection. Moreover, the signal-to-noise ratio varies with the base position. Thus, the method proposed in this study to detect SNPs using GO and SG is feasible. According to the strength of the signal-to-noise ratio, mismatched base positions can be distinguished.

### The Fluorescence Quenching Kinetics of GO for Various SNPs

**FIGURE 2:**
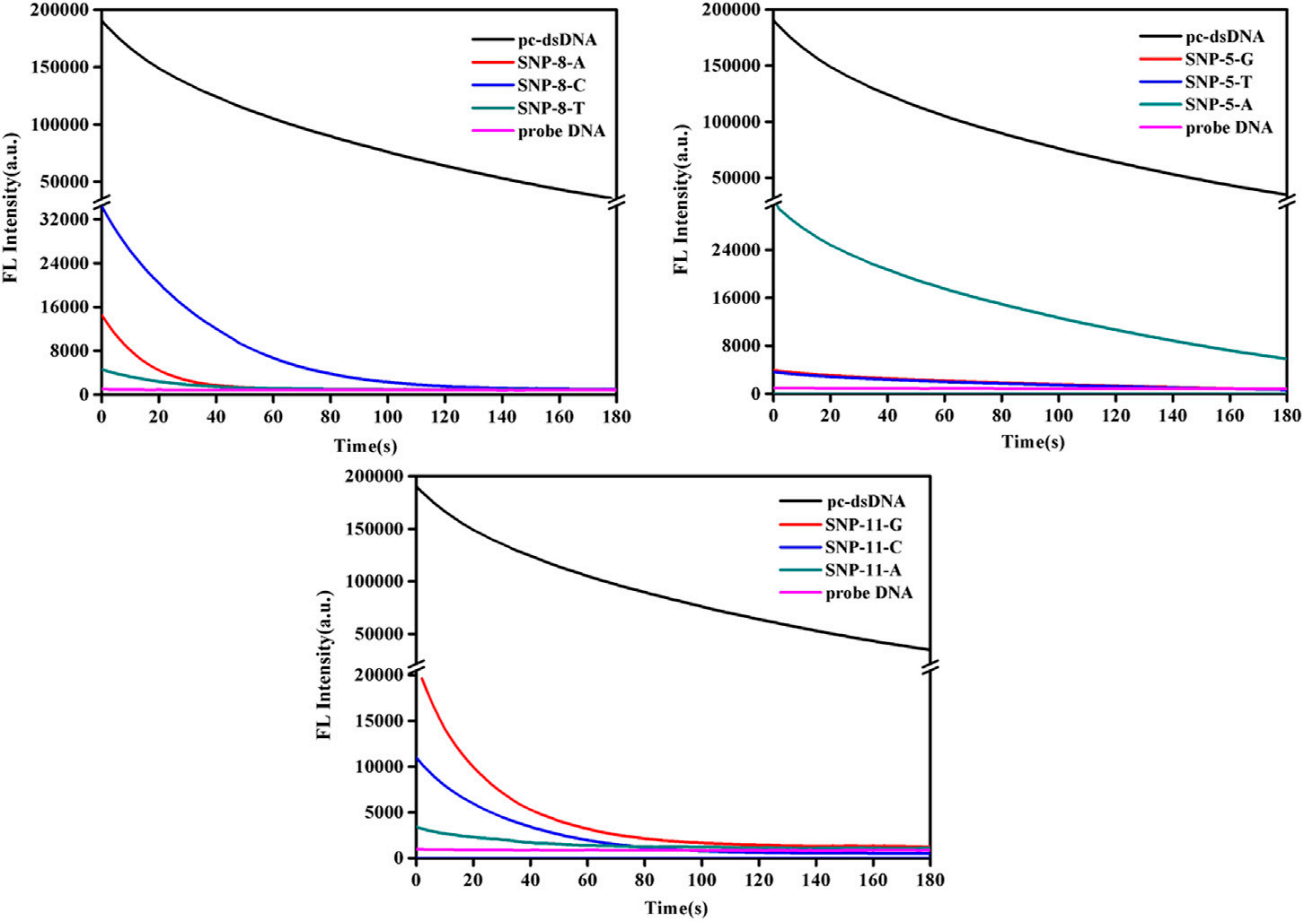
Emission spectra of GO on the detection of SNPs (SNP-8, SNP-5, and SNP-11). The final concentration: [SNPs] = 10 nM, [probe DNA] = 10 nM, [GO] = 2.5 μg/ml, 10 mM phosphate buffer (20 mM NaCl, pH 7.4).

This study further analyzed the dynamic changes of fluorescence values of SNPs with mismatched bases at positions 5, 8, and 11 over time after adding 2.5 μg/ml GO. As shown in Figure 2 and Supplementary Figure S2, the fluorescence quenching of pc-dsDNA was about 80% by GO at 3 min, while that of probe DNA was almost 100% at 15 s, besides, SNP-8, SNP-5, and SNP-11 were completely quenched within 3 min. Supplementary Figure S2 demonstrates that the fluorescence ratios of pc-dsDNA/SNP-8 were between 70 and 100 at 3 min in the present of GO while that of pc-dsDNA/SNP-5 and pc-dsDNA/SNP-11 were 30–50 and 20–80, respectively. Therefore, the fluorescence value was generally detected 15 s after the addition of GO.

### Dynamic Changes of Fluorescence Value of SNPs at Different Salt Concentrations and Temperatures, Respectively

In order to dramatically distinguish SNPs for this sensor, salt concentration and temperature were analyzed. Actually, the two important factors were considered to be closely related to DNA hybridization, SG’s selective intercalation with dsDNA, and GO’s preferential strong adsorption with ssDNA (Swathi and Sebastian, 2009; Liu et al., 2011; Huang and Liu, 2012). The fluorescence values of the samples were all measured at the maximum emission peak *λ* = 525 nm. According to the detection procedure optimized for salt concentration, the following results were obtained. The results in Supplementary Figure S3 show that when the salt concentration was 0.2 mM, pc-dsDNA was best distinguished from probe DNA, and the quenching effect of GO on pc-dsDNA exhibited much slower kinetics and lower efficiency in PBS, and about 80% of the fluorescence of pc-dsDNA was quenched at 3 min. When the salt concentration increased, the fluorescence radio of pc-dsDNA/probe DNA decreased quickly, while The fluorescence quenching was nearly 100% for probe DNA (15 s) in Supplementary Figure S1. This indicates that the GO/probe DNA interaction depends on the concentration of NaCl, and it also indicated that GO strongly adsorbed ssDNA with high affinity, not dsDNA. Besides, the influence of NaCl concentration on GO/SNP interaction was investigated to improve SNP discrimination, as shown in Supplementary Figure S3 that the fluorescence quenching behavior for SNPs (SNP-8, SNP-5, and SNP-11) also depended on NaCl concentration. When the salt concentration was 0.2 mM, F_pc-dsDNA_/F_SNP-8_ (>68) were much higher than F_pc-dsDNA_/F_SNP-5_ (<52), which illustrated that different positions of the smDNAs’ mismatched bases (Supplementary Table S1) had different discrimination, middle position mismatched bases of the smDNAs had the best fluorescence quenching effect within the bases while F_pc-dsDNA_/F_SNP-5-T_ (52) or F_pc-dsDNA_/F_SNP-5-G_ (48) was higher than F_pc-dsDNA_/F_SNP-5-A_ (8) at 0.2 mM NaCl. This shows that different mismatched bases in the same position had also different discrimination. Therefore, SNPs from pcDNA could be easily detected when NaCl concentration at 0.2 mM NaCl, at which point the good F_pc-dsDNA_/F_SNPs_ (>20) was enough for SNP discrimination (Supplementary Table S1).

**FIGURE 3:**
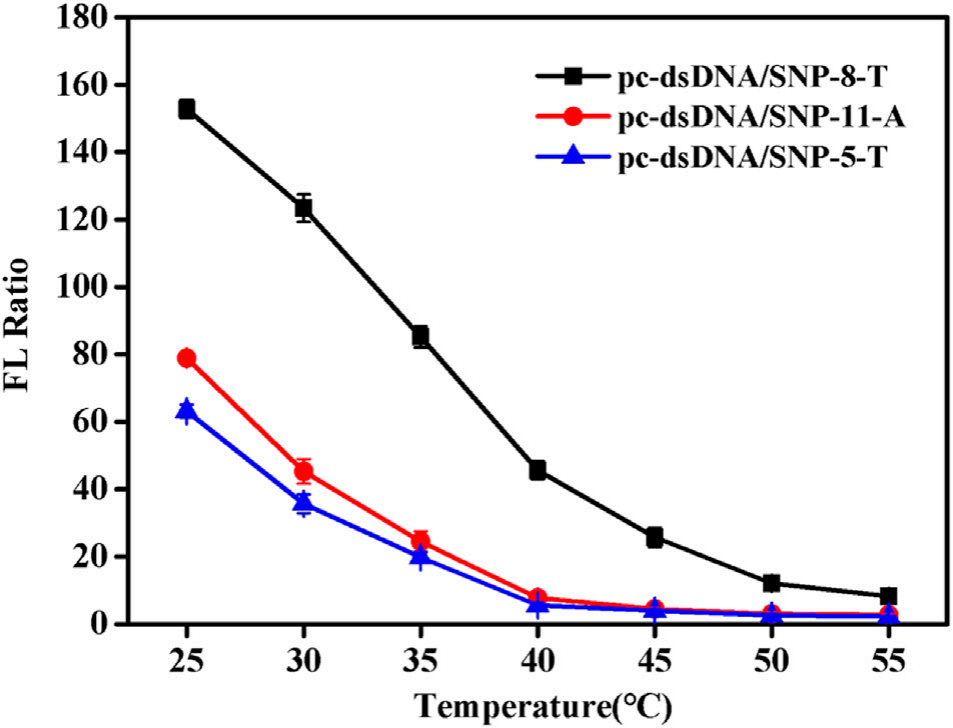
Emission spectra of with varying pc-dsDNA/SNP-5-T, pc-dsDNA/SNP-8-T and pc-dsDNA/SNP-11-A ratios at different temperatures (25, 30, 35, 40, 45, 50, and 55°C). The final concentration: [SNPs] = 10 nM, [pc-dsDNA] = 10 nM, [GO] = 2.5 μg/ml, 10 mM phosphate buffer (20 mM NaCl, pH 7.4). Error bars represented the SD of five experiments.

Since NaCl concentration had a great relationship of the Tm of duplex, the detection temperature at certain salt concentration (0.2 mM) was investigated. Supplementary Figure S4 shows the fluorescence values of the samples at different temperatures. SNP-8-T, SNP-5-T, and SNP-11-A were selected as the representatives of the SNP group. It was discovered that the fluorescence value of SNPs did not change much as the temperature increased. Also, the fluorescence value of pc-dsDNA decreased significantly with the increase of temperature. The difference between the two fluorescence values was greatest at 25°C. Figure 3 shows the fluorescence ratio of pc-dsDNA to SNP-8-T, SNP-5-T, and SNP-11-A at different temperatures. The results show that as the temperature rises, the fluorescence ratio continues to decrease. At 25°C, the ratio of the two was the largest, indicating that the signal-to-noise ratio was very high and the discrimination effect was optimal. These results confirm that the best temperature condition for this monitoring system is 25°C, which means that we can easily differentiate pc-dsDNA from SNPs by this GO-based sensor in a room temperature.

### The Sensitivity of the Biosensor

**FIGURE 4:**
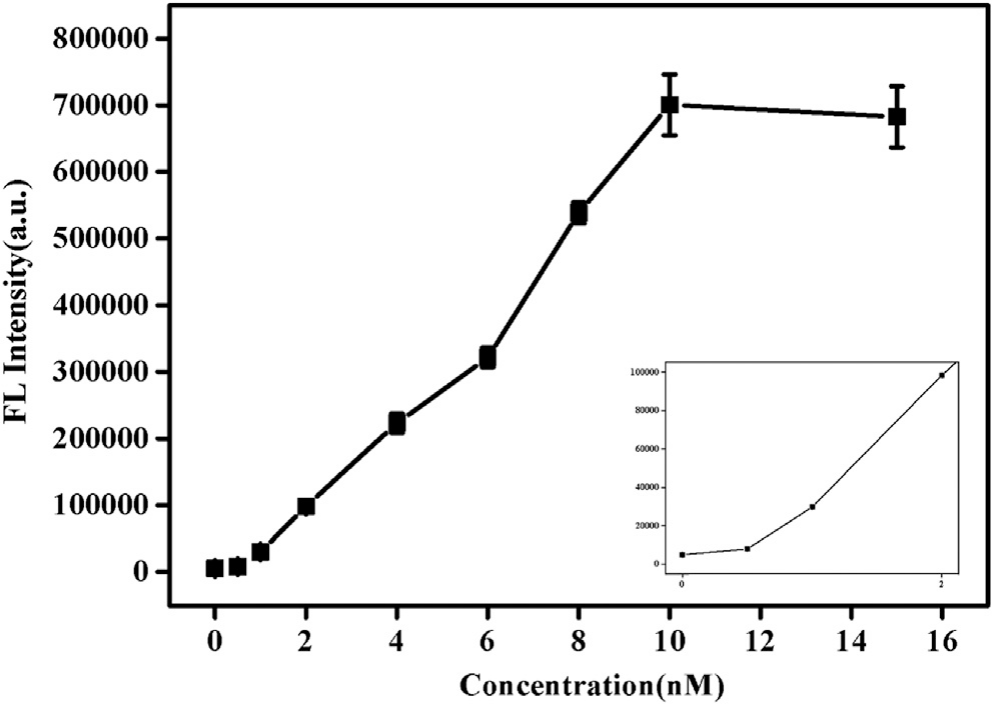
Fluorescence spectra of SG/pc-dsDNA in the presence of various concentrations of pcDNA (0, 0.5, 1, 2, 4, 6, 8, 10, 12, and 15 nM, and the total target DNA containing probe and pcDNA was 20 nM). Other conditions were same as Figure 1.

Under the best experimental conditions, the sensitivity of this method to detect SNPs was analyzed. As shown in Figure 4, with the increase of pcDNA concentration, its fluorescence value also increased. When the concentration reached 10 nM, the fluorescence value hardly changed. Supplementary Figure S5 shows the trend of fluorescence value as a function of pcDNA concentration. Similarly, when the concentration was 10 nM, equilibrium was reached, and the fluorescence value was almost no longer increased. This indicates that enough pcDNA and probe DNA had been hybridized at this time, and the reaction was complete. In addition, based on the results of detection of SNPs reported in previous studies (Supplementary Table S2; Wei et al., 2016; Xu et al., 2018; Wolfe et al., 2019), this paper uses graphene oxide-based SYBR Green I fluorescent intercalating dye-induced sensors to detect SNPs with a low detection limit (1 nM). The response time is faster (15 s).

### Different Allele Frequency Analysis

**FIGURE 5:**
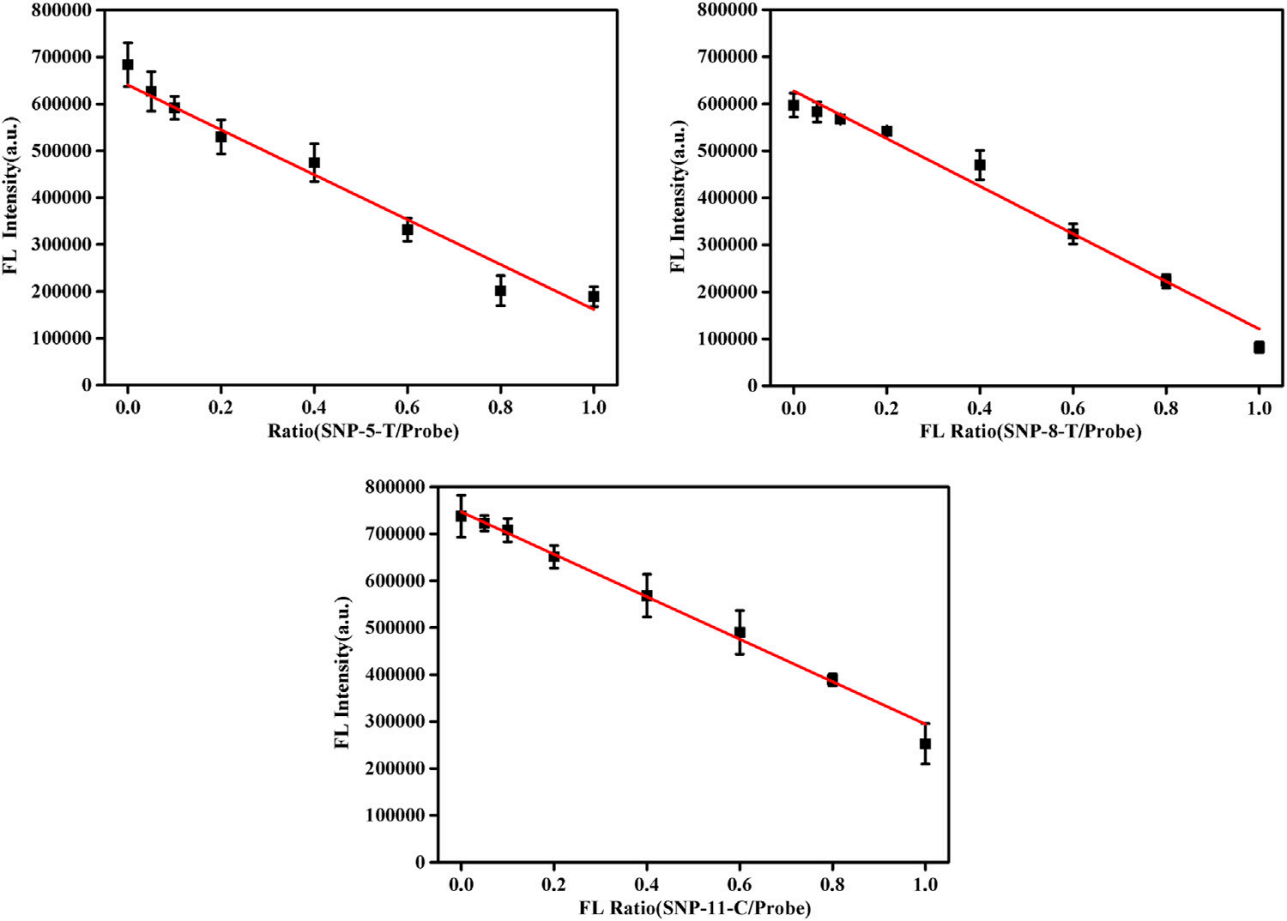
Fluorescence spectra in the presence of different allele frequency [pcDNA/(pcDNA + smDNA) was 0, 5, 10, 20, 40, 60, 80, and 100%, and the total target DNA containing pcDNA and smDNA was 20 nM]. Other conditions were same as Figure 1.

In the proposed detection system, the final concentration of the probe DNA was 10 nM, and the total concentration of pcDNA and the detected smDNA (smDNA-5-T, smDNA-8-T, or smDNA-11-C) was 10 nM. The ratio of pcDNA/(pcDNA + smDNA) was 0, 5, 10, 20, 40, 60, 80, and 100%. Analysis was performed under experimental conditions of 25°C and 0.2 mM NaCl concentration, and the results shown in Figure 5 were obtained. When the ratio of smDNA was 0%, the detected fluorescence value was the largest. As the proportion of smDNA increased, the fluorescence value decreased continuously; when the ratio was 100%, the fluorescence value was the lowest. The graphs in Supplementary Figure S6 show the fluorescence values detected when the ratio of smSNA to target DNA is different with a nice linear gradient, and 5% of SNP target could be differentiated. Obviously, the fluorescence value decreases with the increase of the ratio of mismatched DNA in the target DNA, which fully demonstrates the rationality and feasibility of this method.

## Conclusions

In summary, a new, fast, simple, ultra-highly sensitive and selective biosensor for detecting SNPs was developed here. All above detection results confirm that our biosensor indeed work as we expect. 1) The sensor requires no fluorescent labeling, is simple and inexpensive. 2) SG has specificity for the intercalation strength of each dsDNA, but the specificity is not high enough. Perfectly complementary dsDNA expresses high fluorescence emission due to the high intercalation of SG, while SNP is relatively low. After adding GO, the interaction between GO and SNP is much greater than that between GO and pc-dsDNA. The fluorescence emitted by SG/SNP is quickly quenched by GO, and the fluorescence value is almost zero. However, the fluorescence of SG/pc-dsDNA is still high. At this time, the fluorescence ratio of SG/pc-dsDNA to SG/SNP is up to 120 times, and the signal-to-noise ratio is very high. 3) Under optimized conditions, we could detect 1 nM DNA with 0–10 nM linear range and differentiate 5% SNP, with a response time of only 15 s. The sensor is expected to make a significant contribution to pharmacogenomics and medical diagnosis.

## Data Availability

The datasets presented in this study can be found in online repositories. The names of the repository/repositories and accession number(s) can be found in the article/Supplementary material.
