# Supplementary material for: Detection of Single Nucleotide Polymorphisms by Fluorescence Embedded Dye SYBR Green I Based on Graphene Oxide
Source: Front Chem. 2021 Mar 31;9:631959. doi: 10.3389/fchem.2021.631959 (PMC8044317; doi:10.3389/fchem.2021.631959)
Supplement: Supplementary file 1 [file datasheet1.zip › Supplementary material.pdf]

Supplementary Material

Detection of single nucleotide Polymorphisms by fluorescence embedded Dye SYBR Green I based on graphene oxide

Jiaoyun Xia[1], Tong Xu[1], Jing Qing[1], Lihua Wang[2]， Junlong Tang[3*]

[1]School of Chemistry and Food Engineering, Changsha University of Science and Technology, Changsha, China

[2]Shanghai Institute of Applied Physics, Chinese Academy of Sciences, Shanghai, China

[3]School of Physics and Electronic Science, Changsha University of Science and Technology, Changsha, China

*** Correspondence:**
Corresponding Author
Tangjl625@163.com

Contents

Figure S1. Emission spectra of a biosensor consisting of graphene oxide (GO) and SYBR Green I (SG) that detects single nucleotide polymorphisms (SNPs).

Figure S2. Emission spectra of GO on the detection of SNPs (SNP-8, SNP-5 and SNP-11).

Figure S3. Emission spectra of with varying pc-dsDNA/SNP-8, pc-dsDNA/SNP-5 and pc-dsDNA/SNP-11 ratios in different salt concentrations.

Figure S4. Emission spectra of with varying pc-dsDNA/SNP-5-T, pc-dsDNA/SNP-8-T and pc-dsDNA/SNP-11-A ratios at different temperatures (25°C, 30°C, 35°C, 40°C, 45°C, 50°C and 55°C).

Figure S5. Fluorescence spectra of SG/pc-dsDNA in the presence of various concentrations of pcDNA (0, 0.5, 1, 2, 4, 6, 8, 10, 12 and 15 nM), and the total target DNA containing probe and pcDNA was 20 nM.

Figure S6. Fluorescence spectra in the presence of different allele frequency (pcDNA/(pcDNA+smDNA) was 0%, 5%, 10%, 20%, 40%, 60%, 80% and 100%, and the total target DNA containing pcDNA and smDNA was 20 nM).

Table S1. Complementary and single-base mismatch capture oligonucleotides.

Table S2. Comparison of analysis methods for detecting SNPs.

1 Supplementary Figures

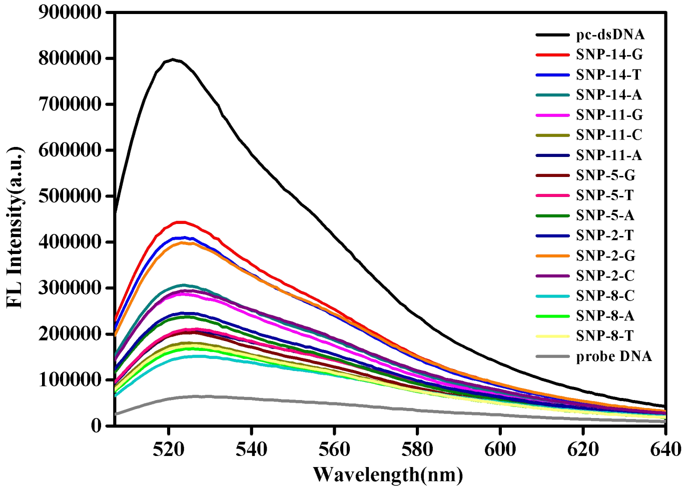

Figure S1 Emission spectra of a biosensor consisting of graphene oxide (GO) and SYBR Green I (SG) that detects single nucleotide polymorphisms (SNPs). The final concentration: [SNPs] = 10 nM, [probe DNA] = 10 nM, [pcDNA] = 10 nM, [GO] = 2.5 μg/mL, 10 mM phosphate buffer (20 mM NaCl, pH 7.4). λ_{ex} = 497 nm, λ_{em} = 525 nm.

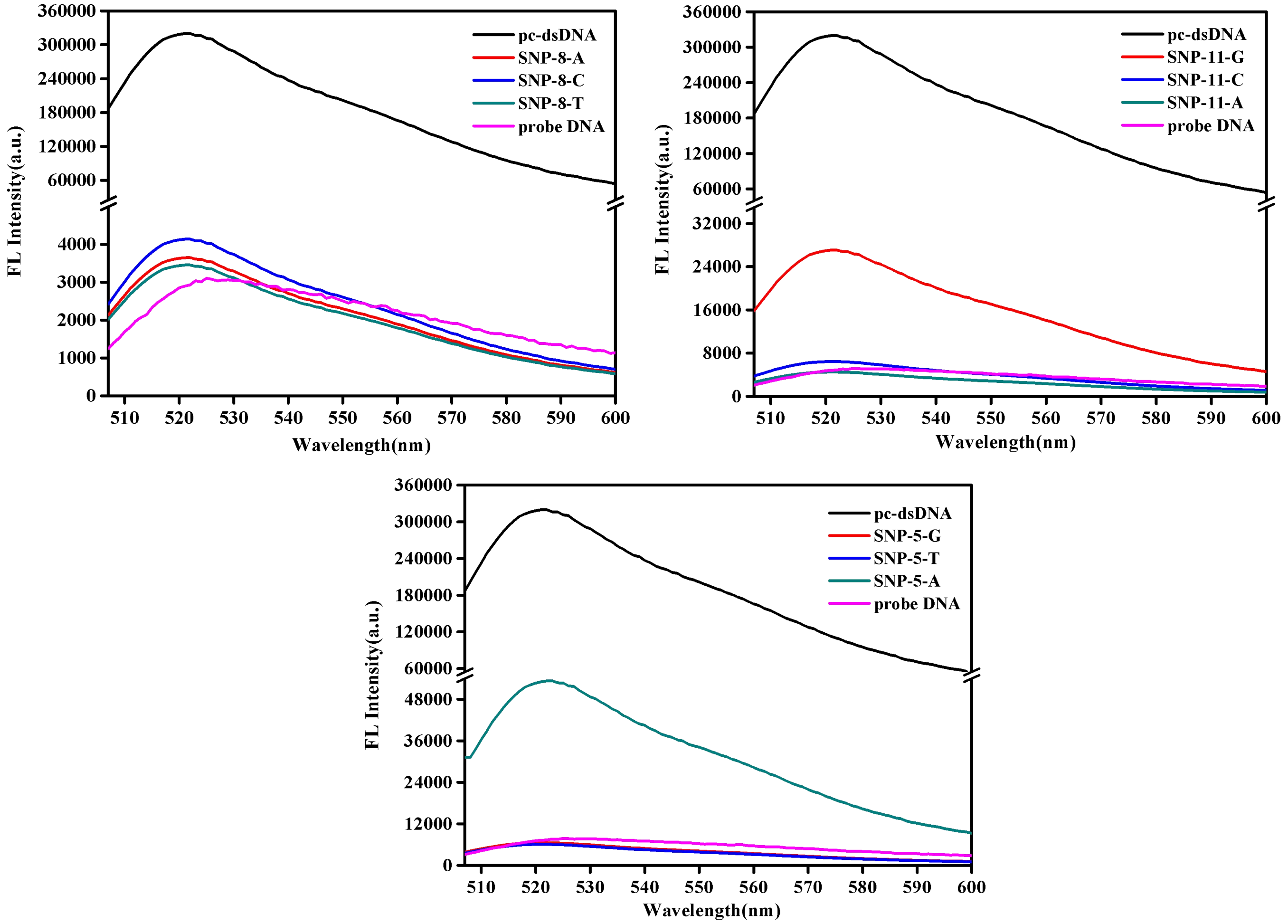

Figure S2 Emission spectra of GO on the detection of SNPs (SNP-8, SNP-5 and SNP-11). The final concentration: [SNPs] = 10 nM, [probe DNA] = 10 nM, [GO] = 2.5 μg/mL, 10 mM phosphate buffer (20 mM NaCl, pH 7.4).

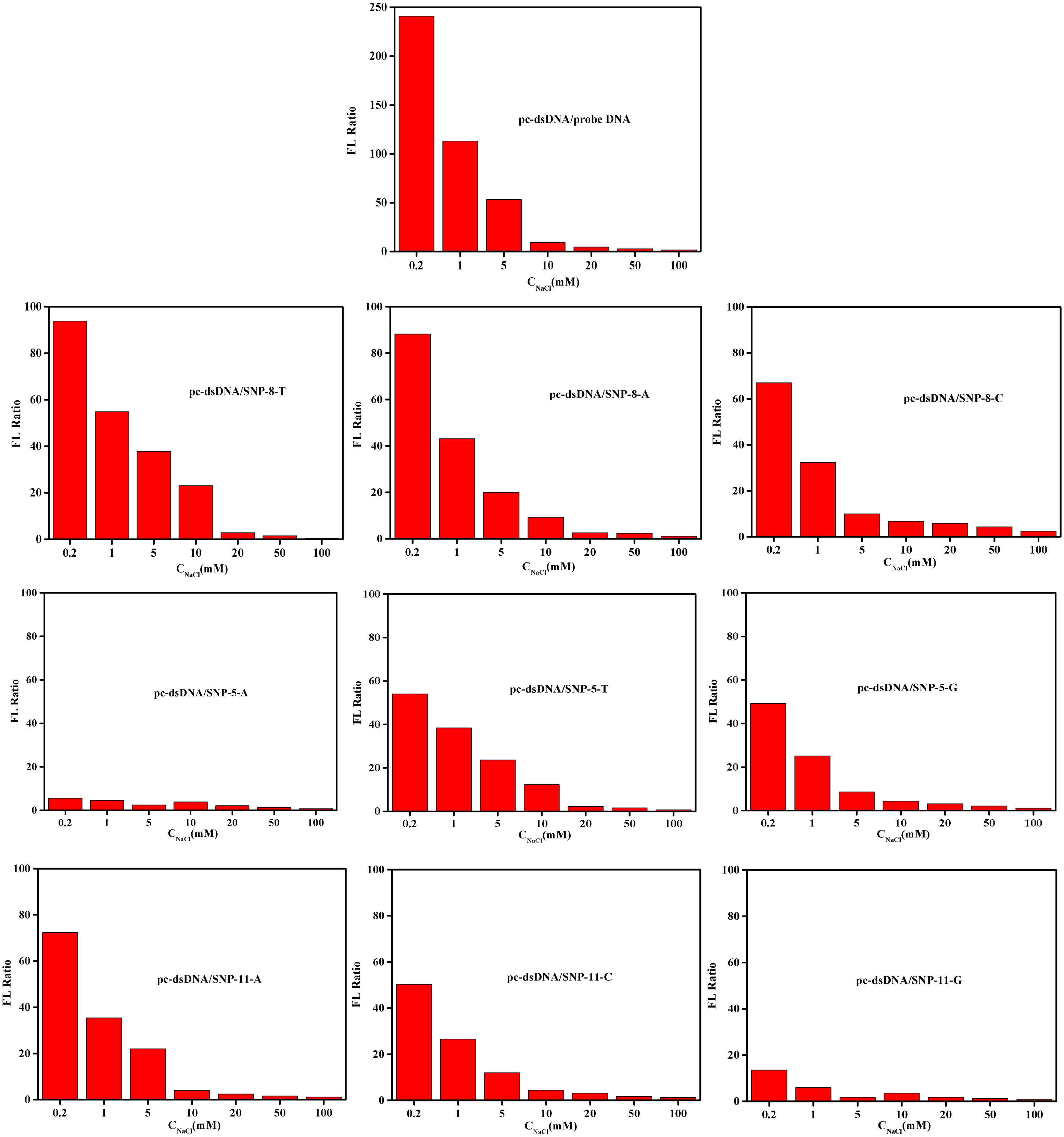

Figure S3 Emission spectra of with varying pc-dsDNA/SNP-8, pc-dsDNA/SNP-5 and pc-dsDNA/SNP-11 ratios in different salt concentrations. The final concentration: [SNPs] = 10 nM, [pc-dsDNA] = 10 nM, [GO] = 2.5 μg/mL, 10 mM phosphate buffer (0.2, 1.0, 5.0, 10, 20, 50 and 100 mM NaCl, pH 7.4).

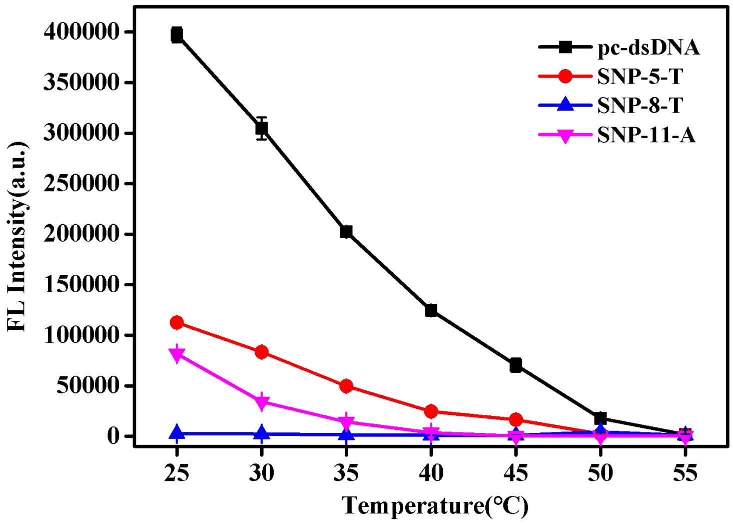

Figure S4 Emission spectra of with varying pc-dsDNA/SNP-5-T, pc-dsDNA/SNP-8-T and pc-dsDNA/ SNP-11-A ratios at different temperatures (25°C, 30°C, 35°C 40°C, 45°C, 50°C and 55°C). The final concentration: [SNPs] = 10 nM, [pc-dsDNA] = 10 nM, [GO] = 2.5 µg/mL, 10 mM phosphate buffer (20 mM NaCl, pH 7.4).

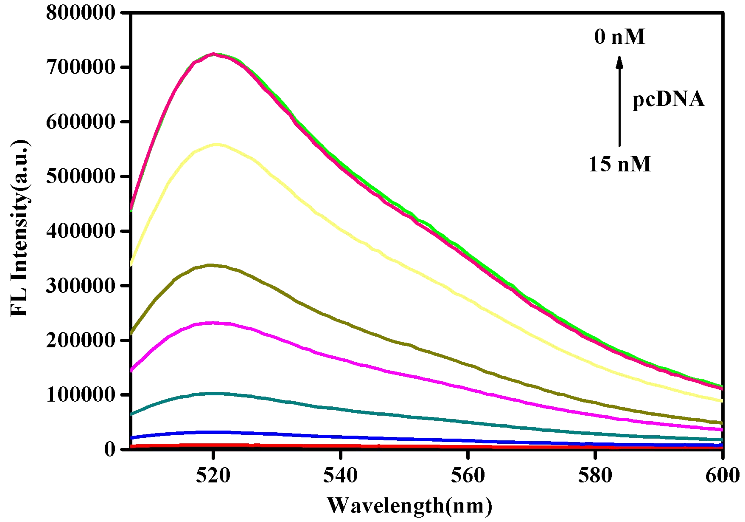

Figure S5 Fluorescence spectra of SG/pc-dsDNA in the presence of various concentrations of pcDNA (0, 0.5, 1, 2, 4, 6, 8, 10, 12 and 15 nM), and the total target DNA containing probe and pcDNA was 20 nM. Other conditions were same as Figure S1.

.

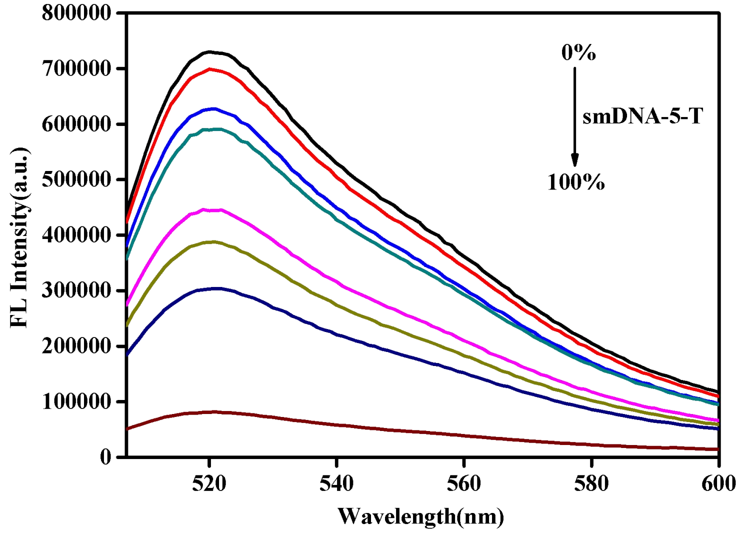

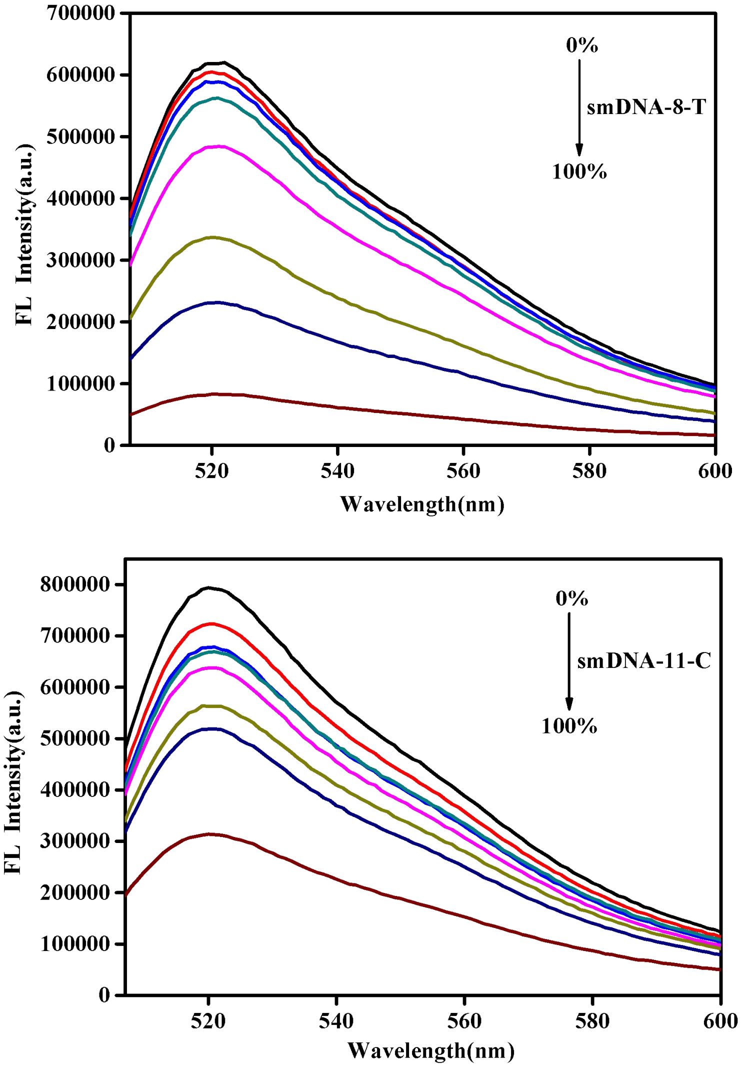

Figure S6 Fluorescence spectra in the presence of different allele frequency (pcDNA/(pcDNA+smDNA) was 0%, 5%, 10%, 20%, 40%, 60%, 80% and 100%, and the total target DNA containing pcDNA and smDNA was 20 nM). Other conditions were same as Figure S1.

2 Supplementary Table

Table S1 Complementary and single-base mismatch capture oligonucleotides

name	Sequence (5'-3 ')
probe DNA	5'-TGCGAACCAGGAATT-3'
pcDNA	5'-AATTCCTGGTTCGCA-3'
sm-DNA-8-T	5'-AATTCCTTGTTCGCA-3'
sm-DNA-8-A	5'-AATTCCTAGTTCGCA-3'
sm-DNA-8-C	5'-AATTCCTCGTTCGCA-3'
sm-DNA-2-C	5'-ACTTCCTGGTTCGCA-3'
sm-DNA-2-G	5'-AGTTCCTGGTTCGCA-3'
sm-DNA-2-T	5'-ATTTCCTGGTTCGCA-3'
sm-DNA-5-A	5'-AATTACTGGTTCGCA-3'
sm-DNA-5-T	5'-AATTTCTGGTTCGCA-3'
sm-DNA-5-G	5'-AATTGCTGGTTCGCA-3'
sm-DNA-11-A	5'-AATTCCTGGTACGCA-3'
sm-DNA-11-C	5'-AATTCCTGGTCCGCA-3'
sm-DNA-11-G	5'-AATTCCTGGTGCGCA-3'
sm-DNA-14-A	5'-AATTCCTGGTTCGAA-3'
sm-DNA-14-T	5'-AATTCCTGGTTCGTA-3'
sm-DNA-14-G	5'-AATTCCTGGTTCGGA-3'

Table S2 Comparison of analysis methods for detecting SNPs

Methods	LOD (nM)	Time (min)	Reference
D/L-Tryptophan guided DNA probe	10 nM	60 min	[1]
colorimetric detection	11 nM	40 min	[2]
SWNT−PNA-based SNP sensor	60 nM	30 min	[3]
SG-GO-biosensor	1 nM	0.25 min	this work

[1] Wei, B., Zhang, T., Ou, X., Li, X., Lou, X., and Xia, F. (2016). Stereochemistry-guided DNA probe for single nucleotide polymorphisms analysis. *Acs Applied Materials & Interfaces* 8(25), 15911-15916. doi:10.1021/acsami.6b03896

[2] Wolfe, M. G., Ali, M. M., and Brennan, J. D. (2019). Enzymatic Litmus Test for Selective Colorimetric Detection of C-C Single Nucleotide Polymorphisms. *Analytical Chemistry* 91(7), 4735-4740. doi:10.1021/acs.analchem.9b00235

[3] Xu, W., Xing, S., Xu, X., Xu, M., Fu, P., Gao, T., et al. (2018). Peptide Nucleic Acid-Assisted Label-free Detection of Single-Nucleotide Polymorphisms Based on Light Scattering of Carbon Nanotubes. *Acs Omega* 3(12), 17835-17841. doi:10.1021/acsomega.8b02655
