# Supplementary figures and images for: Detection of Single Nucleotide Polymorphisms by Fluorescence Embedded Dye SYBR Green I Based on Graphene Oxide
Source: Front Chem. 2021 Mar 31;9:631959. doi: 10.3389/fchem.2021.631959 (PMC8044317; doi:10.3389/fchem.2021.631959)

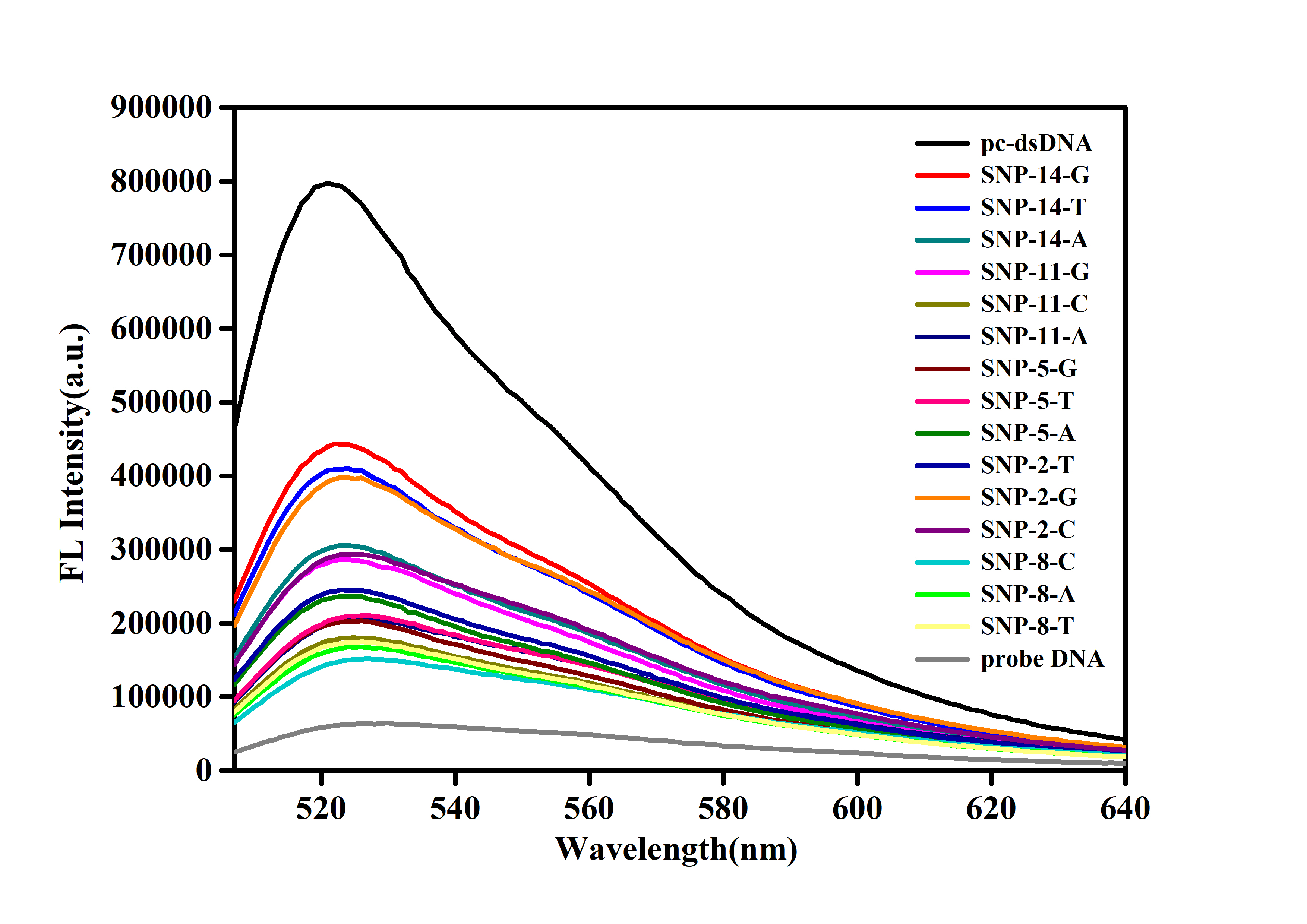

Supplement: Supplementary file 1 [file datasheet1.zip › Supplementary Figures/S1.jpg]

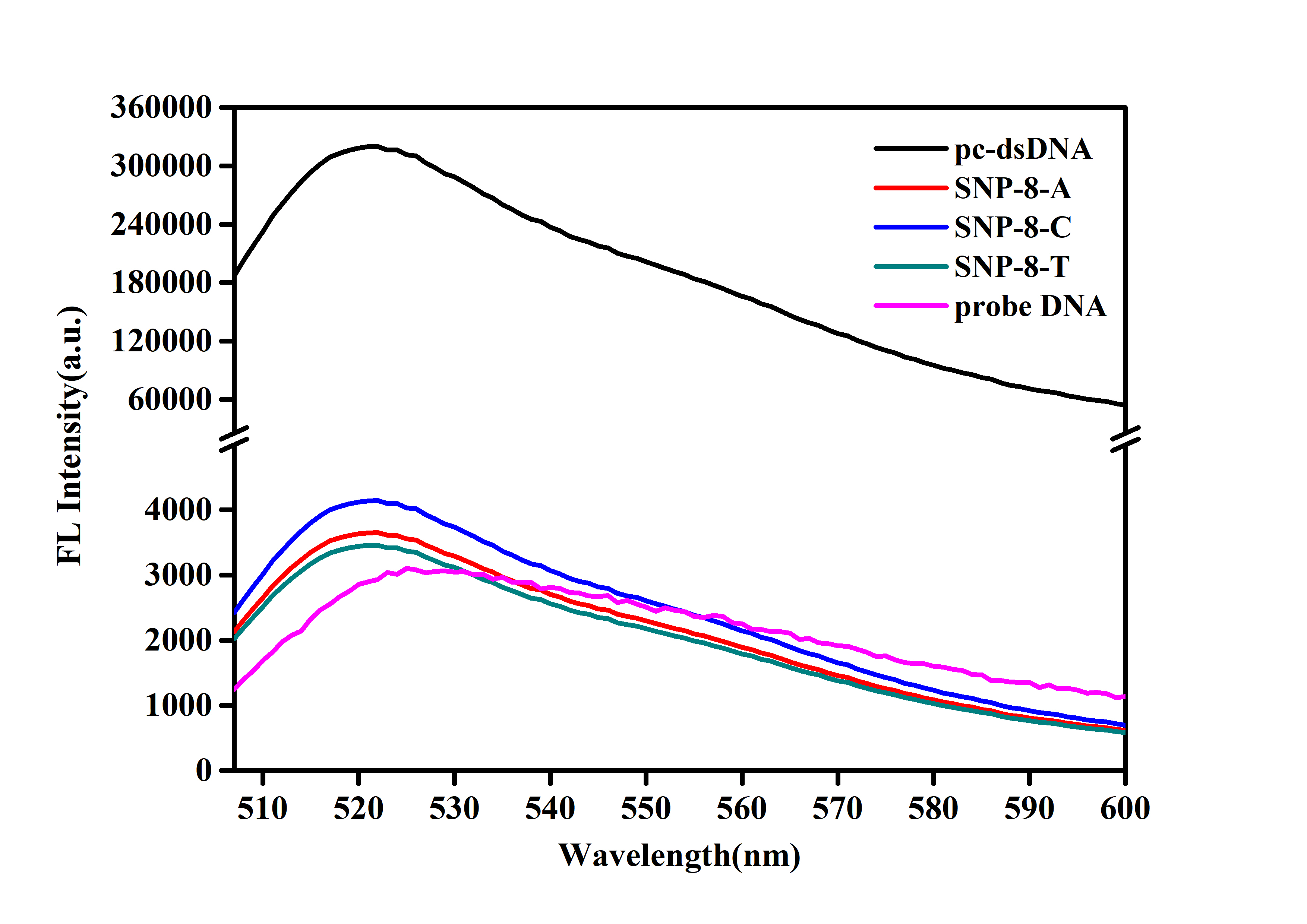

Supplement: Supplementary file 1 [file datasheet1.zip › Supplementary Figures/S2-1.jpg]

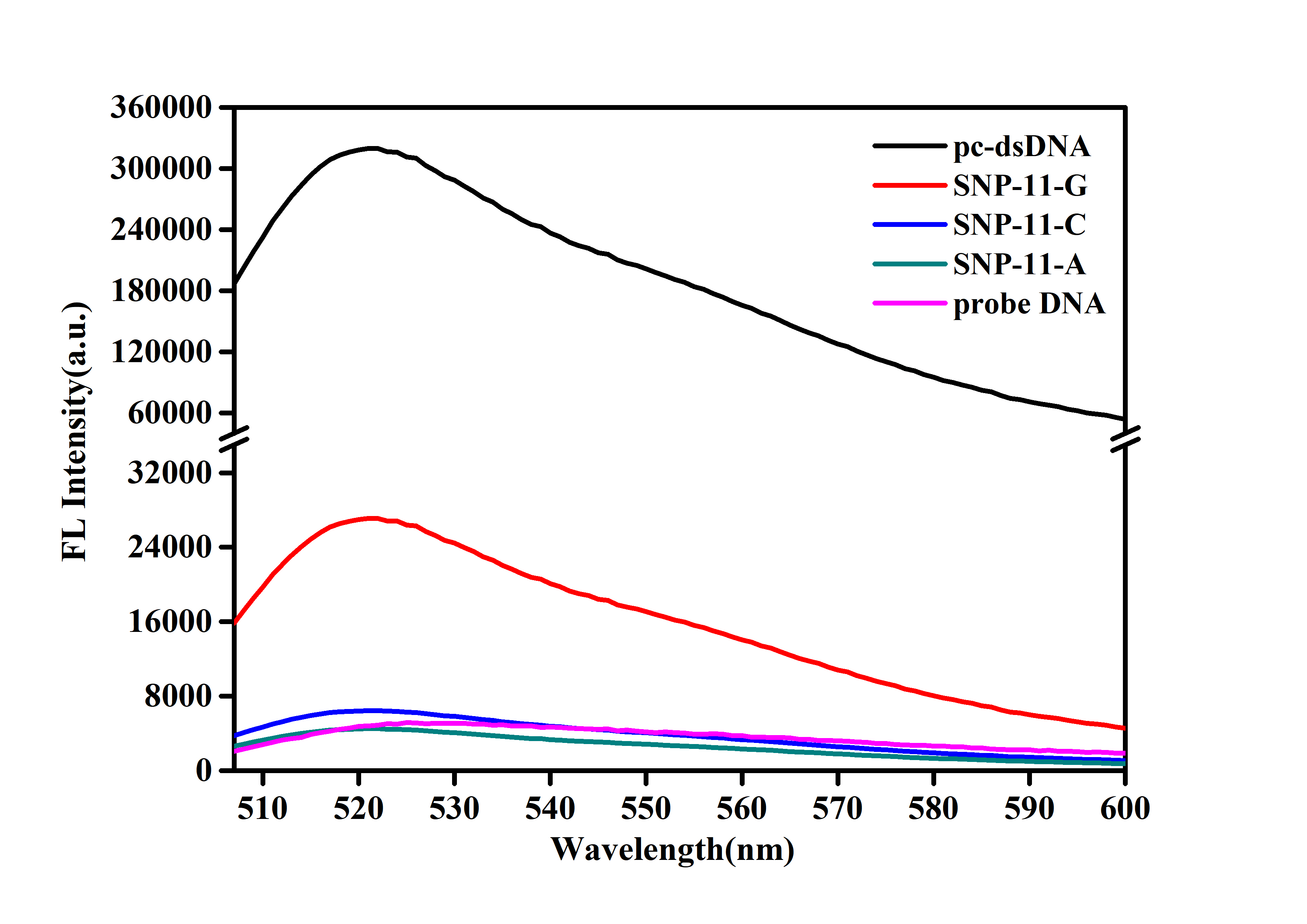

Supplement: Supplementary file 1 [file datasheet1.zip › Supplementary Figures/S2-2.jpg]

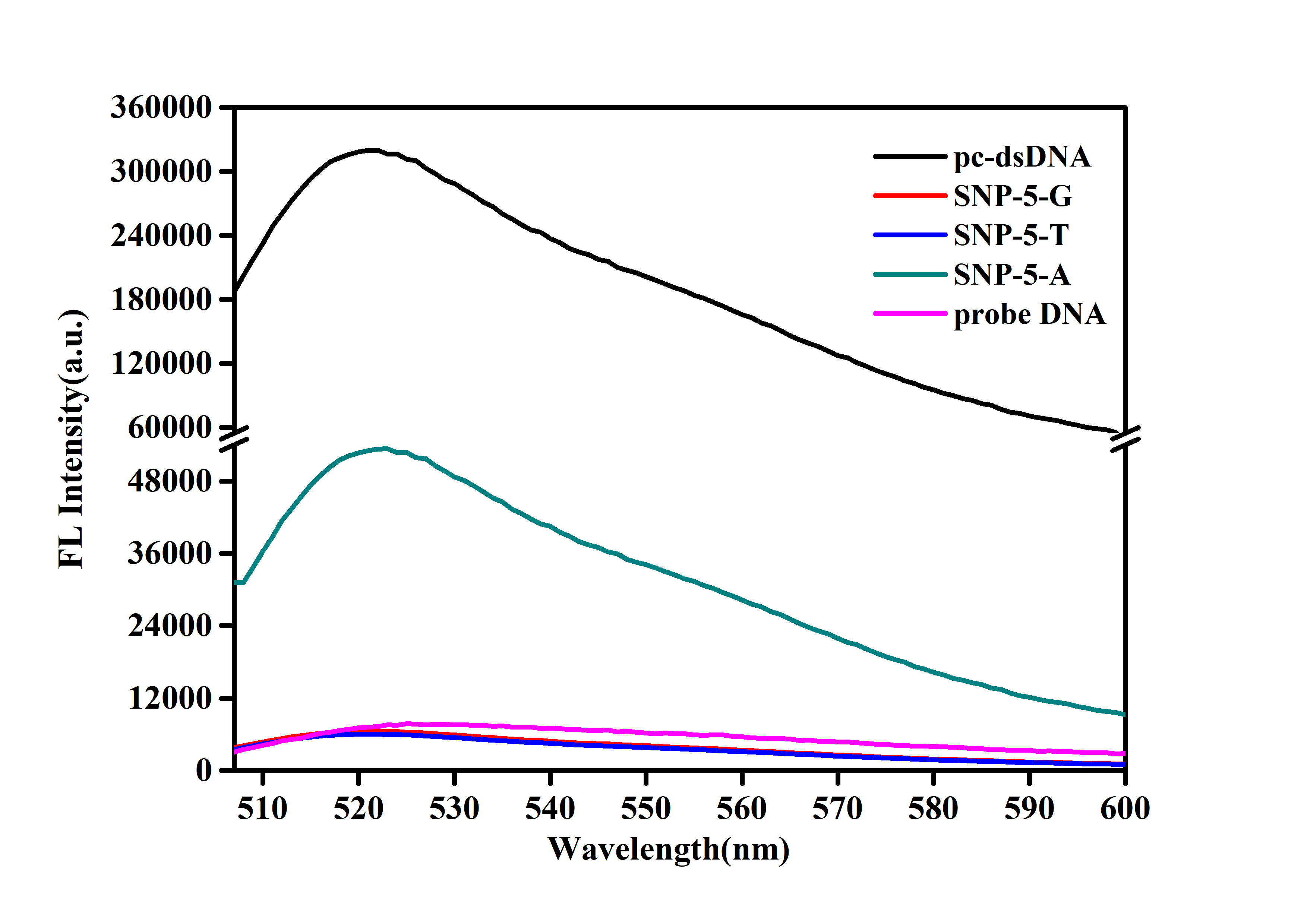

Supplement: Supplementary file 1 [file datasheet1.zip › Supplementary Figures/S2-3.jpg]

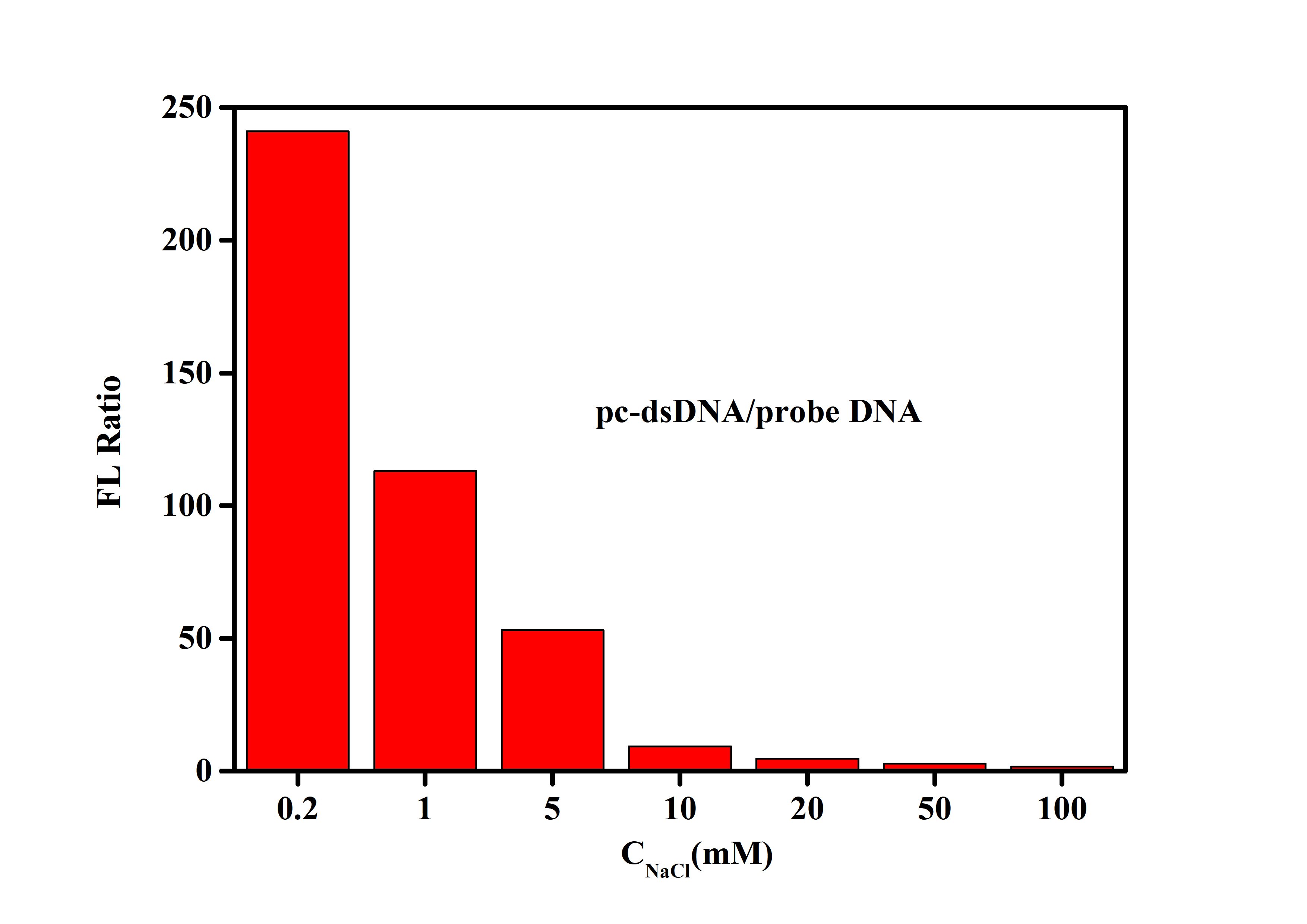

Supplement: Supplementary file 1 [file datasheet1.zip › Supplementary Figures/S3-1.jpg]

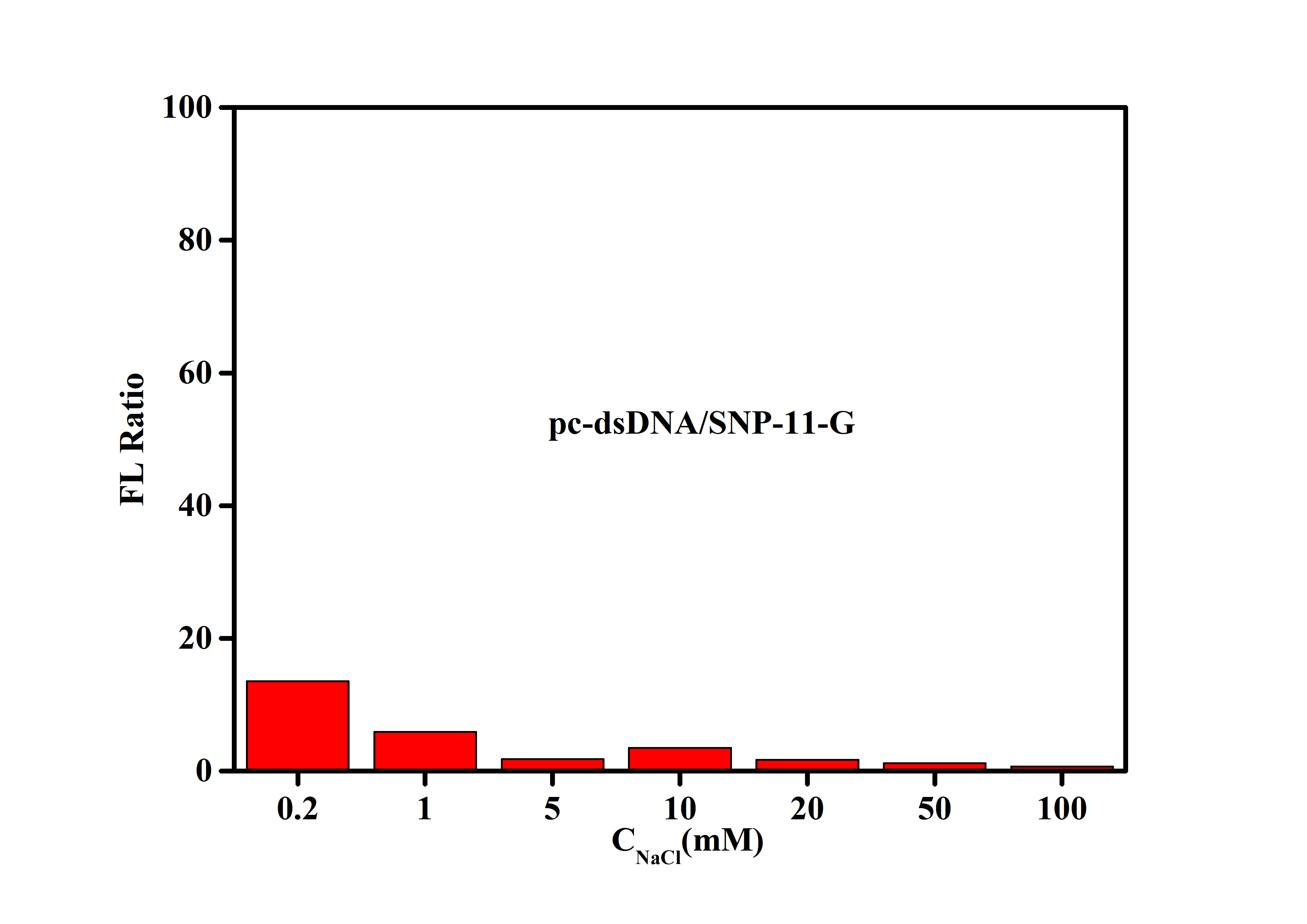

Supplement: Supplementary file 1 [file datasheet1.zip › Supplementary Figures/S3-10.jpg]

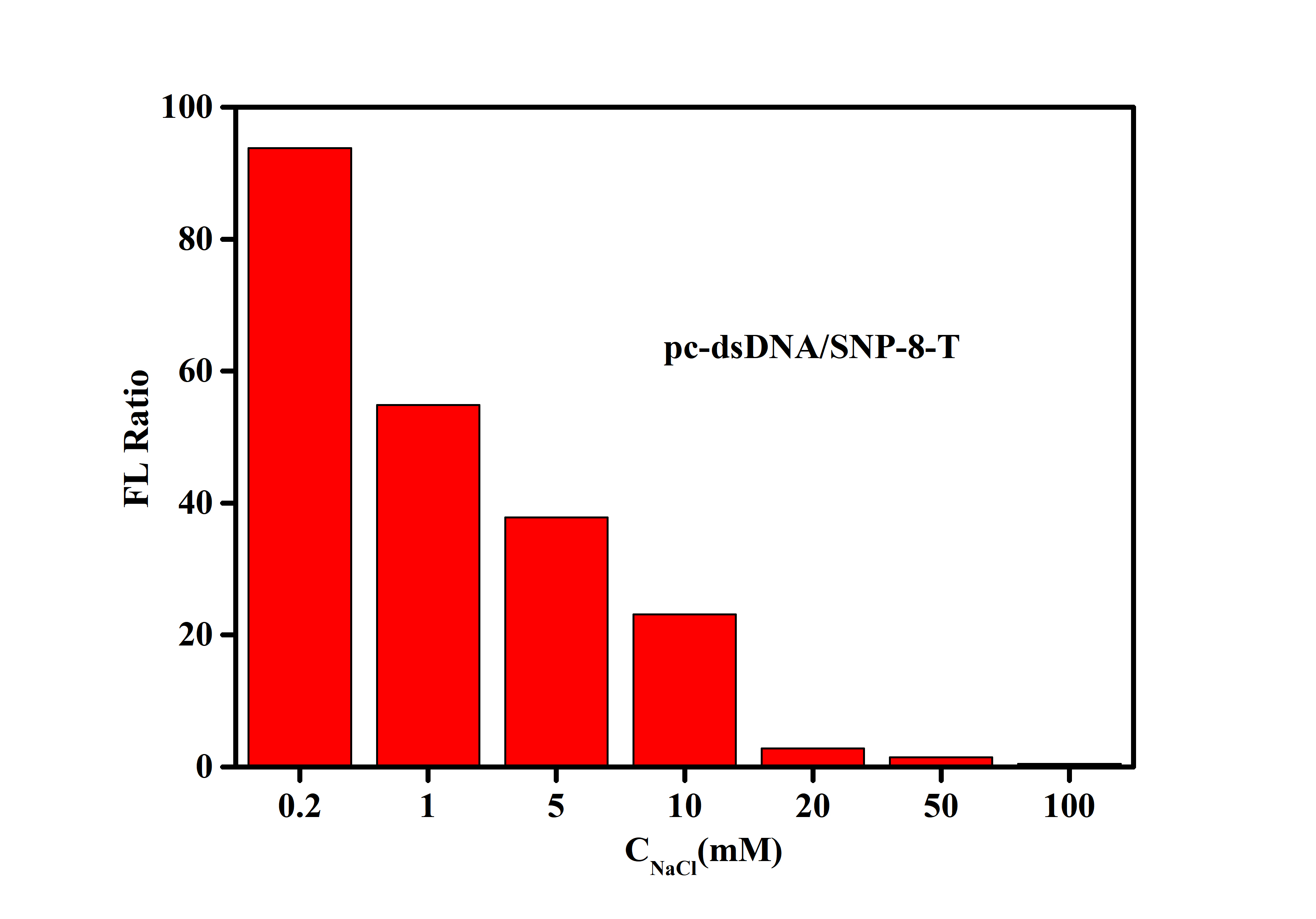

Supplement: Supplementary file 1 [file datasheet1.zip › Supplementary Figures/S3-2.jpg]

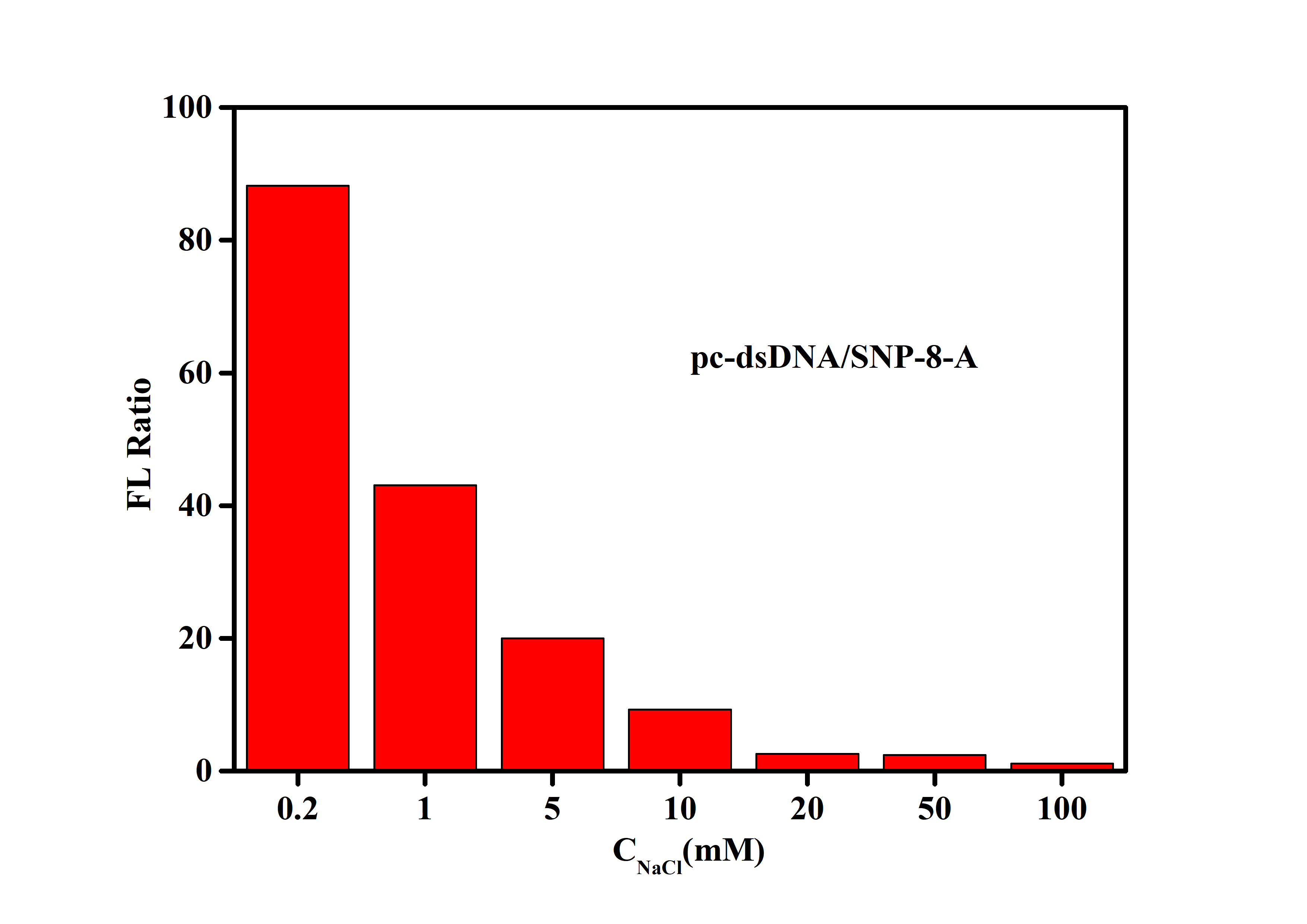

Supplement: Supplementary file 1 [file datasheet1.zip › Supplementary Figures/S3-3.jpg]

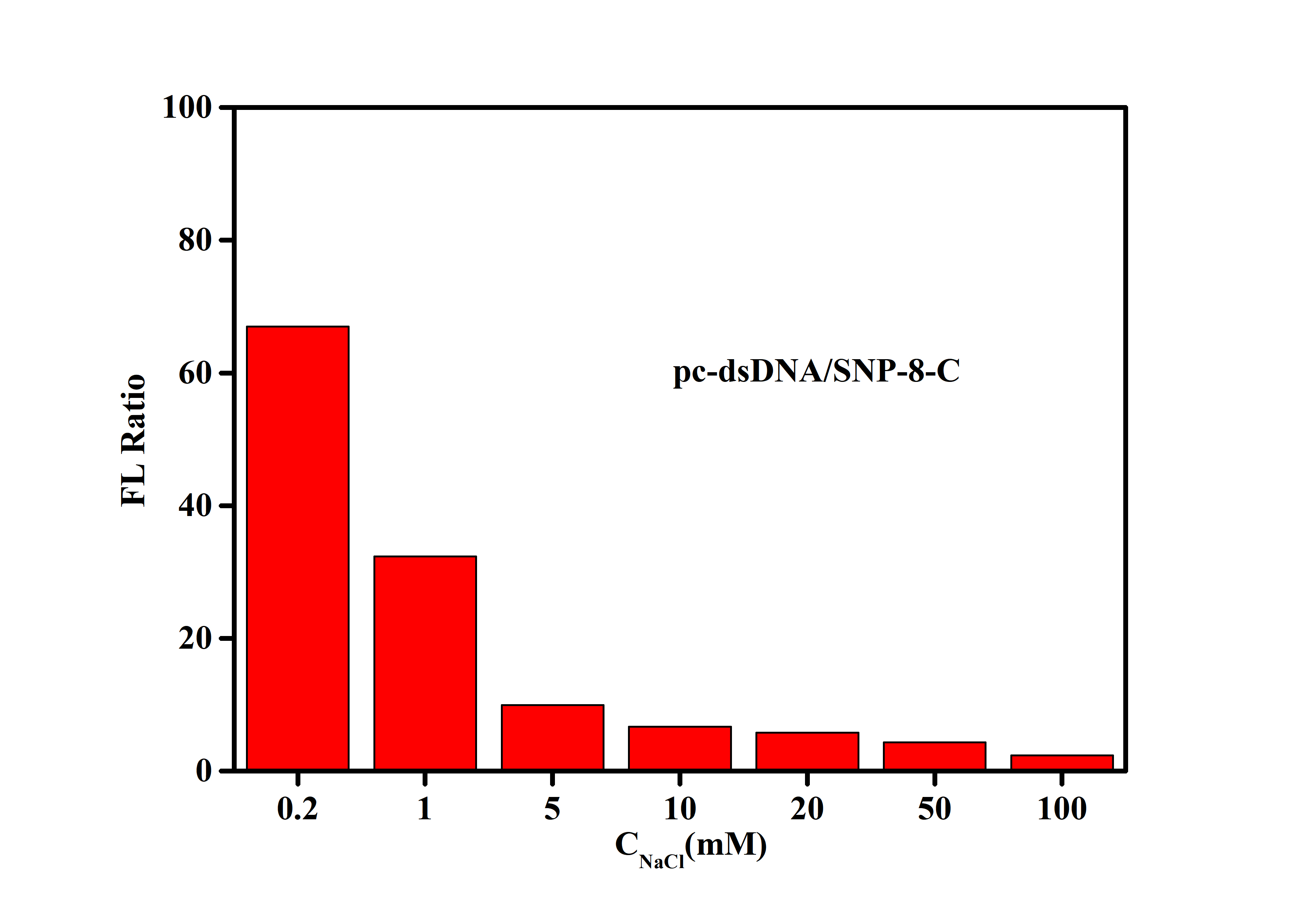

Supplement: Supplementary file 1 [file datasheet1.zip › Supplementary Figures/S3-4.jpg]

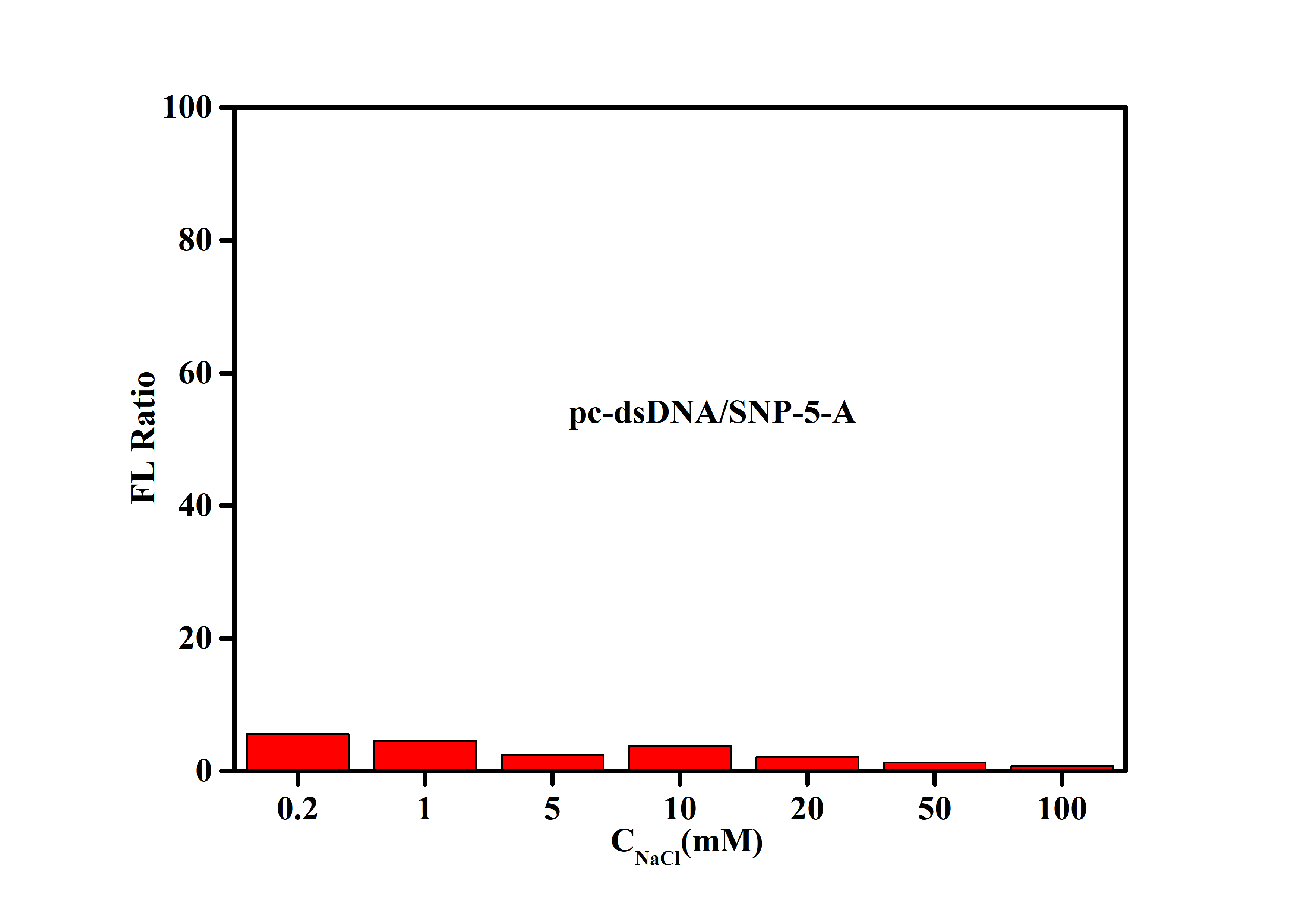

Supplement: Supplementary file 1 [file datasheet1.zip › Supplementary Figures/S3-5.jpg]

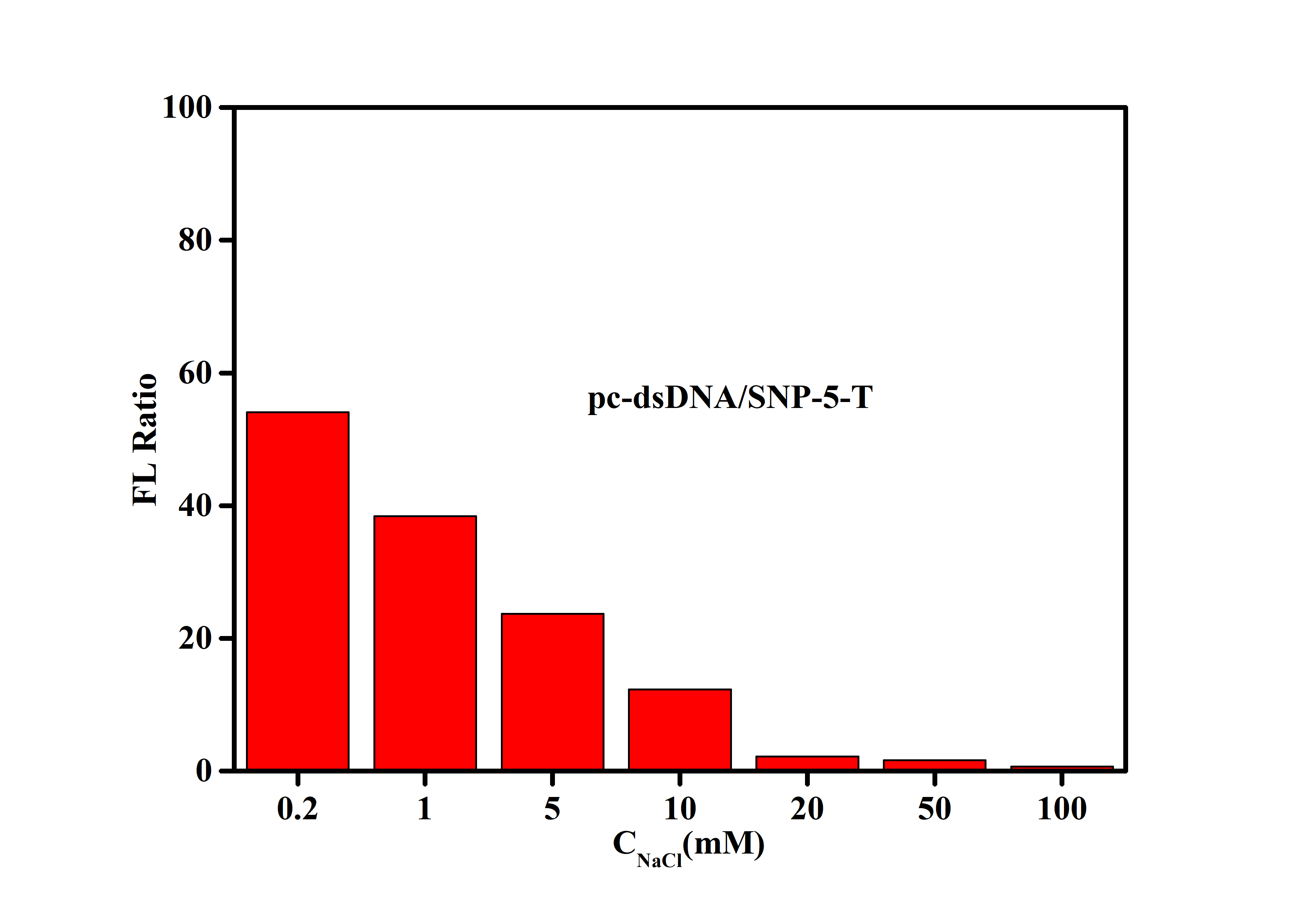

Supplement: Supplementary file 1 [file datasheet1.zip › Supplementary Figures/S3-6.jpg]

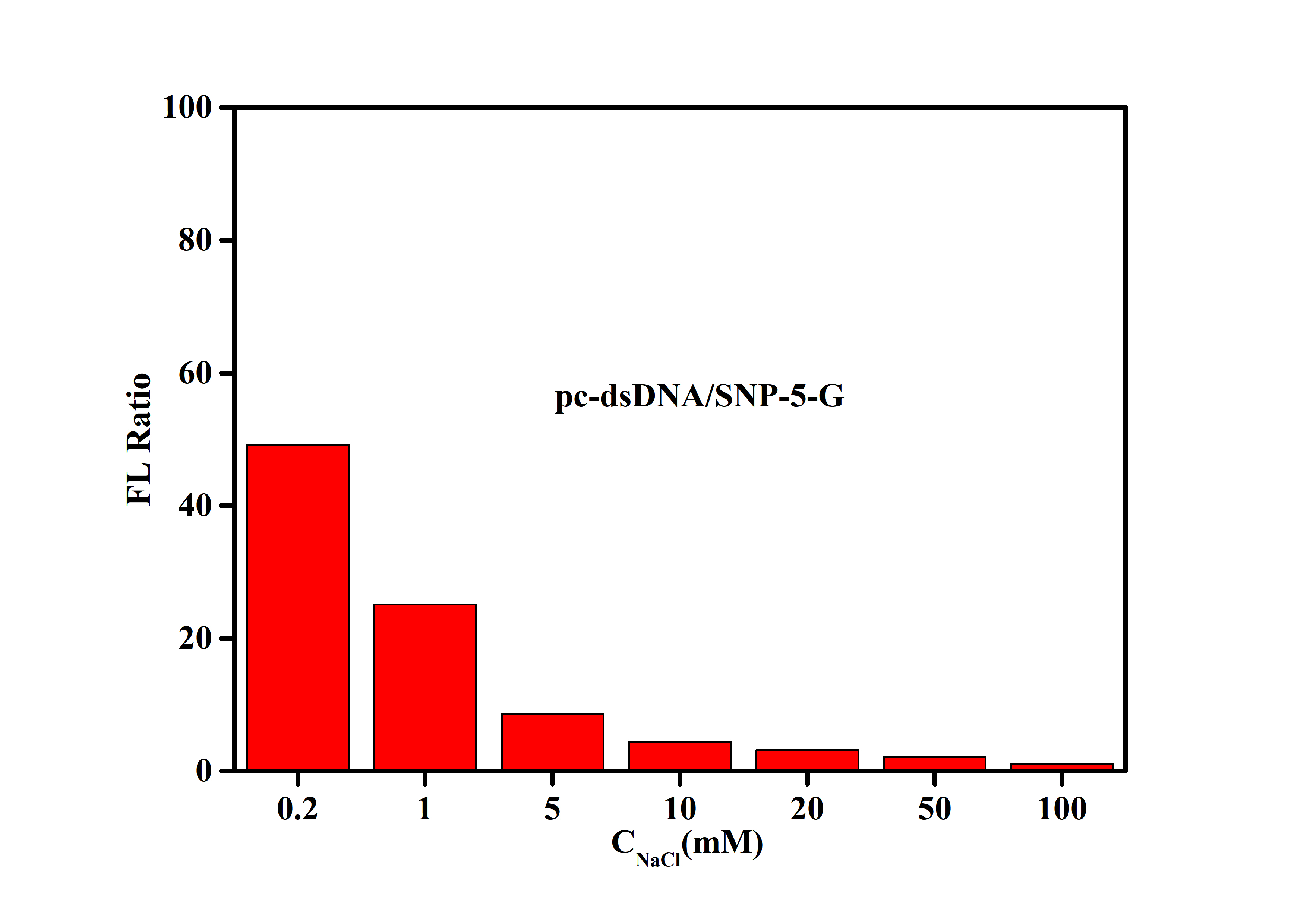

Supplement: Supplementary file 1 [file datasheet1.zip › Supplementary Figures/S3-7.jpg]

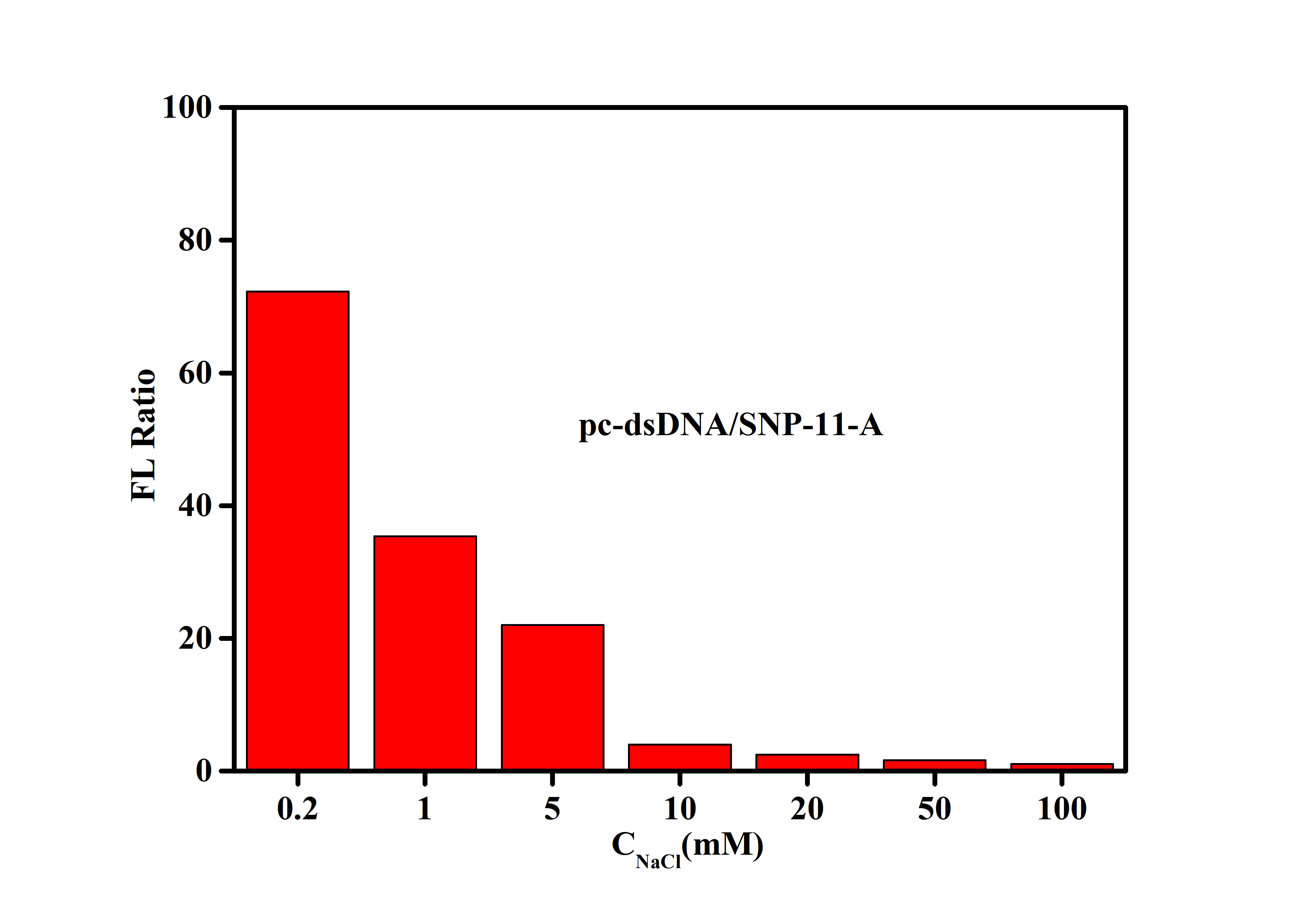

Supplement: Supplementary file 1 [file datasheet1.zip › Supplementary Figures/S3-8.jpg]

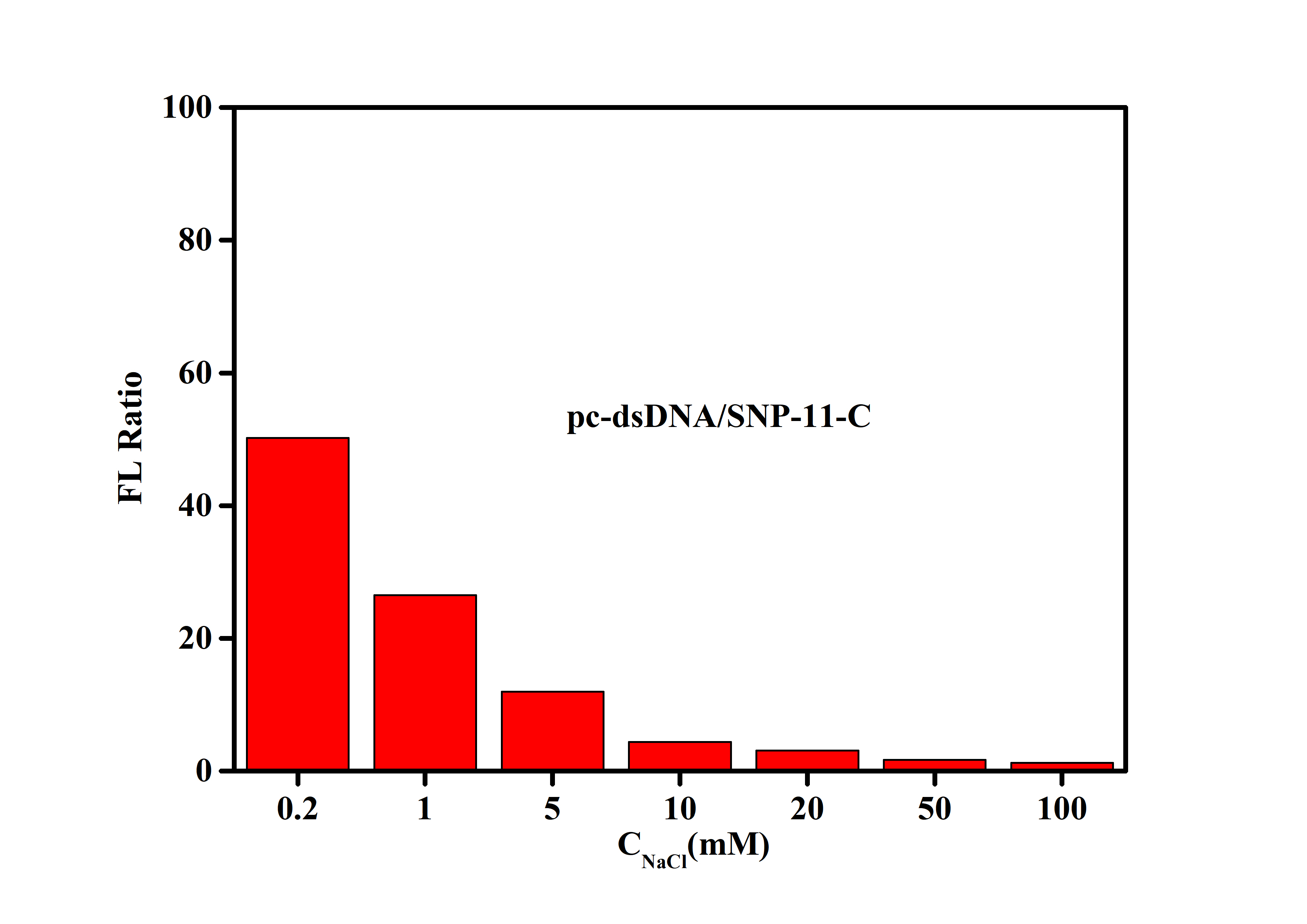

Supplement: Supplementary file 1 [file datasheet1.zip › Supplementary Figures/S3-9.jpg]

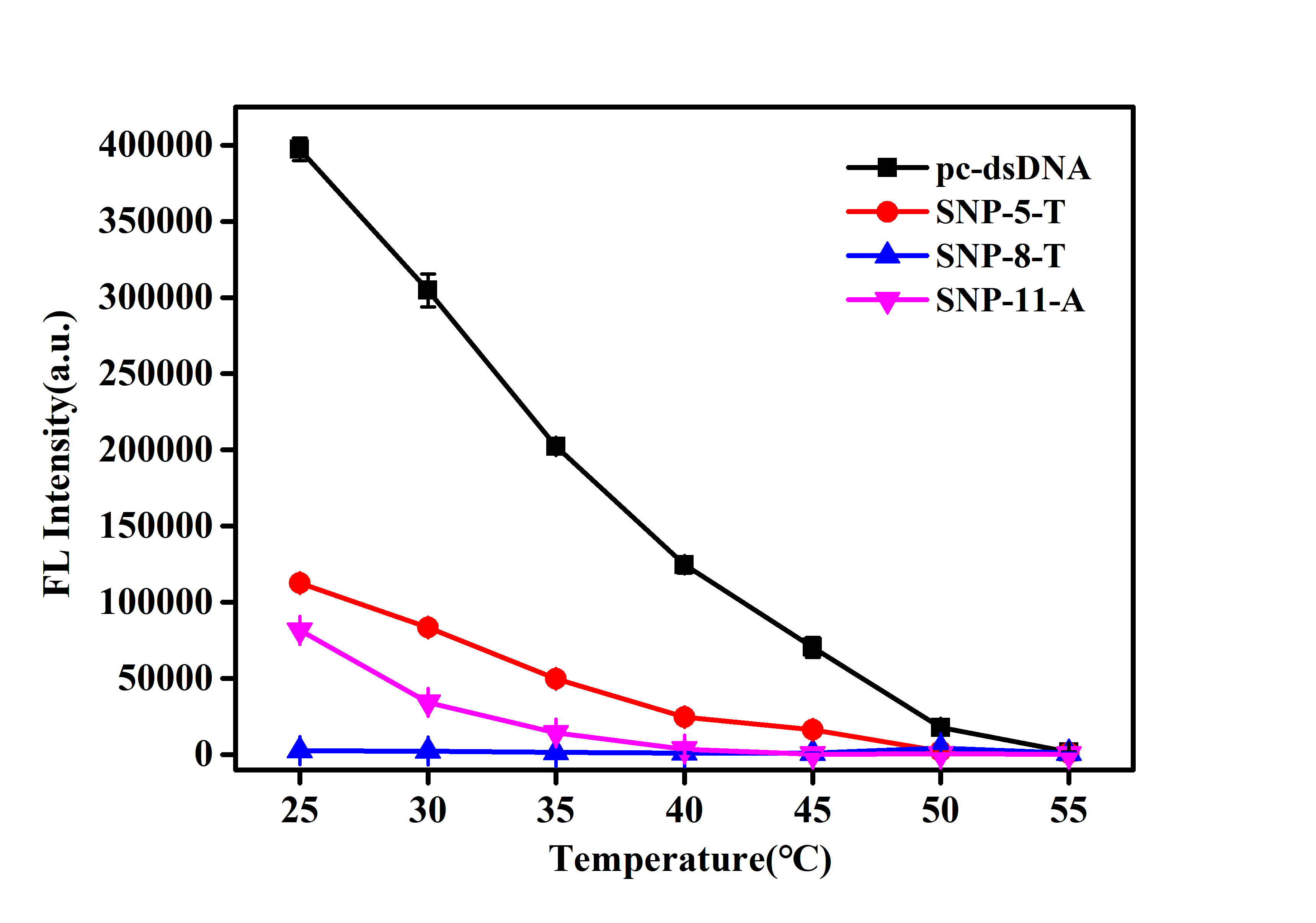

Supplement: Supplementary file 1 [file datasheet1.zip › Supplementary Figures/S4.jpg]

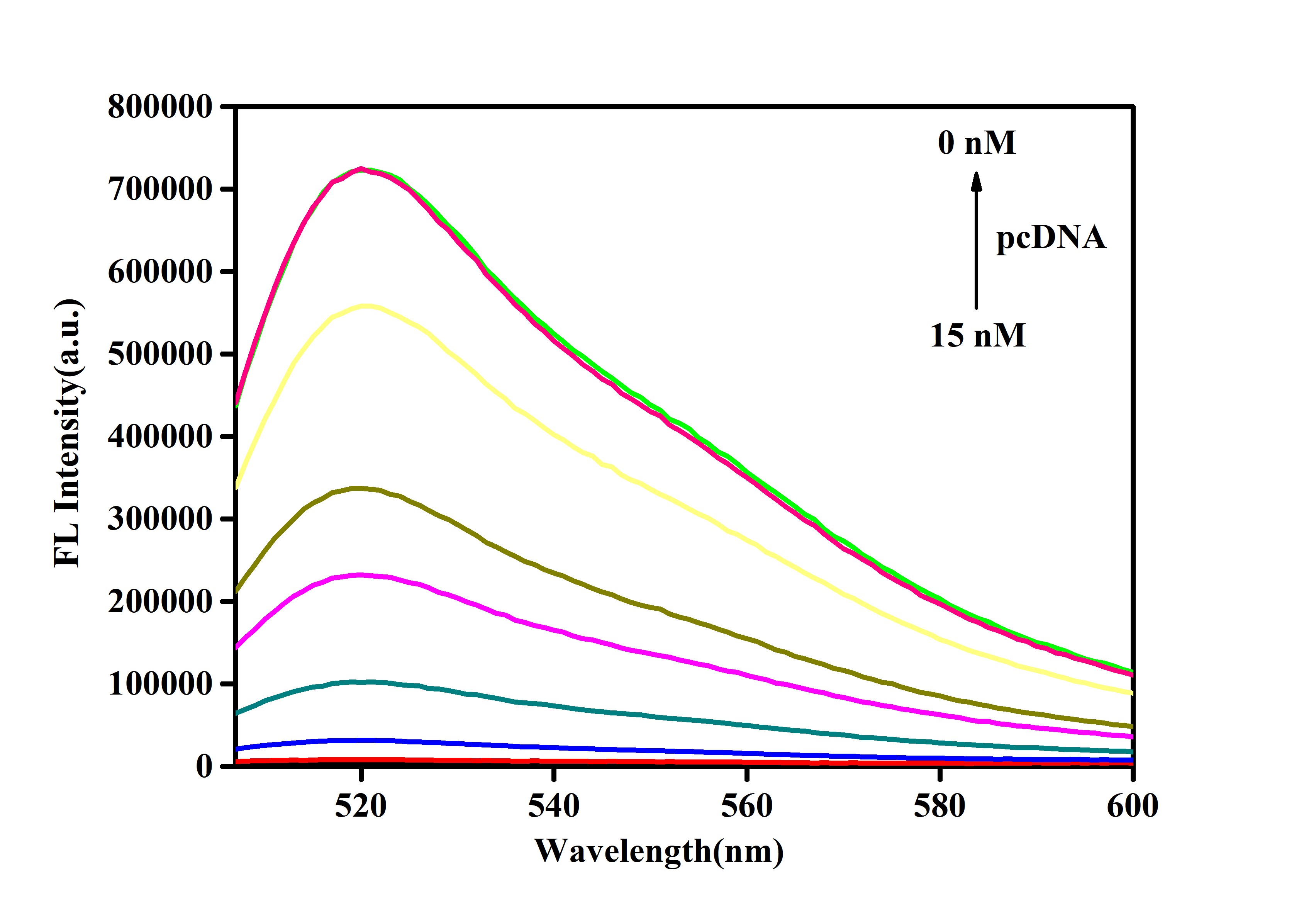

Supplement: Supplementary file 1 [file datasheet1.zip › Supplementary Figures/S5.jpg]

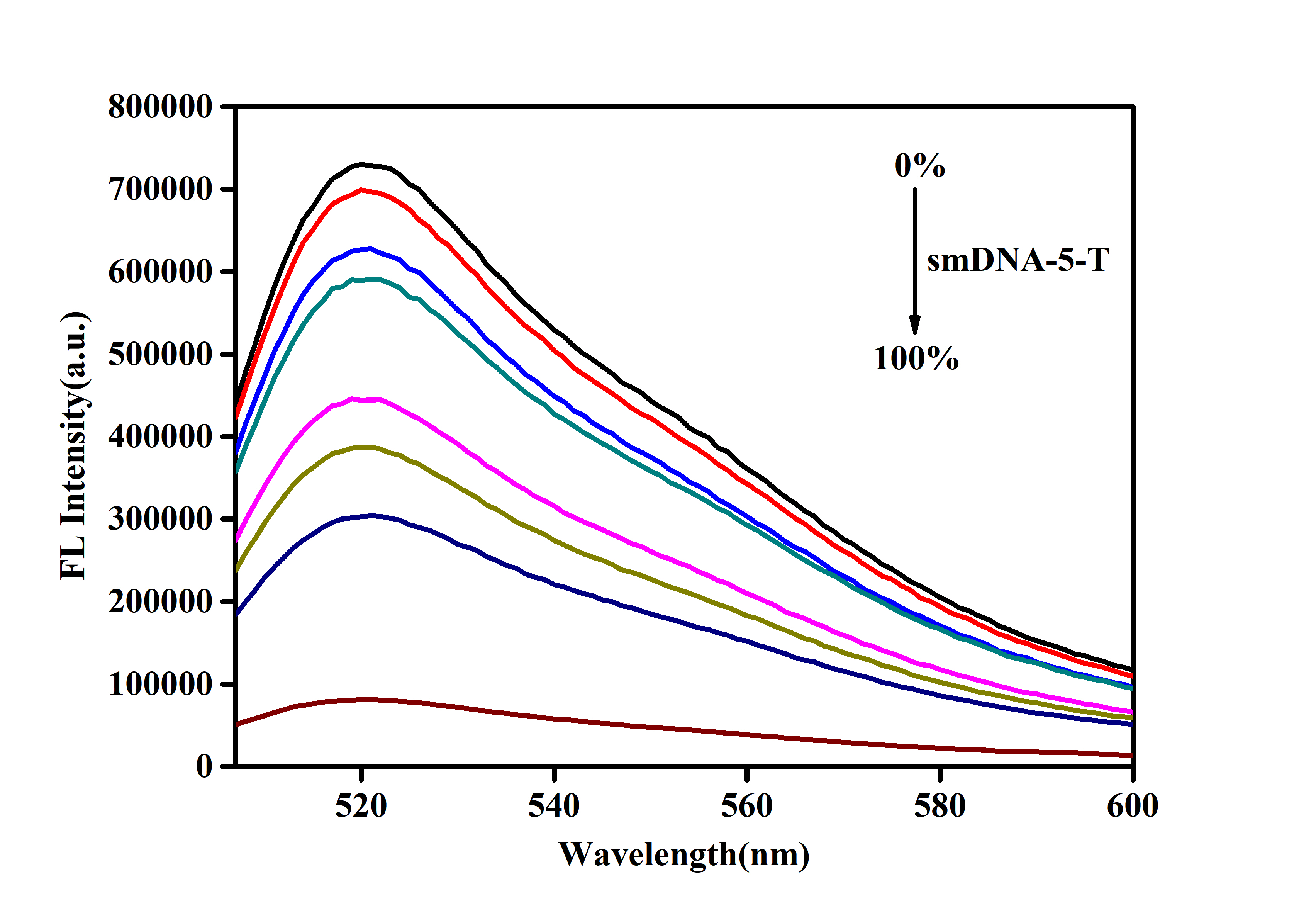

Supplement: Supplementary file 1 [file datasheet1.zip › Supplementary Figures/S6-1.jpg]

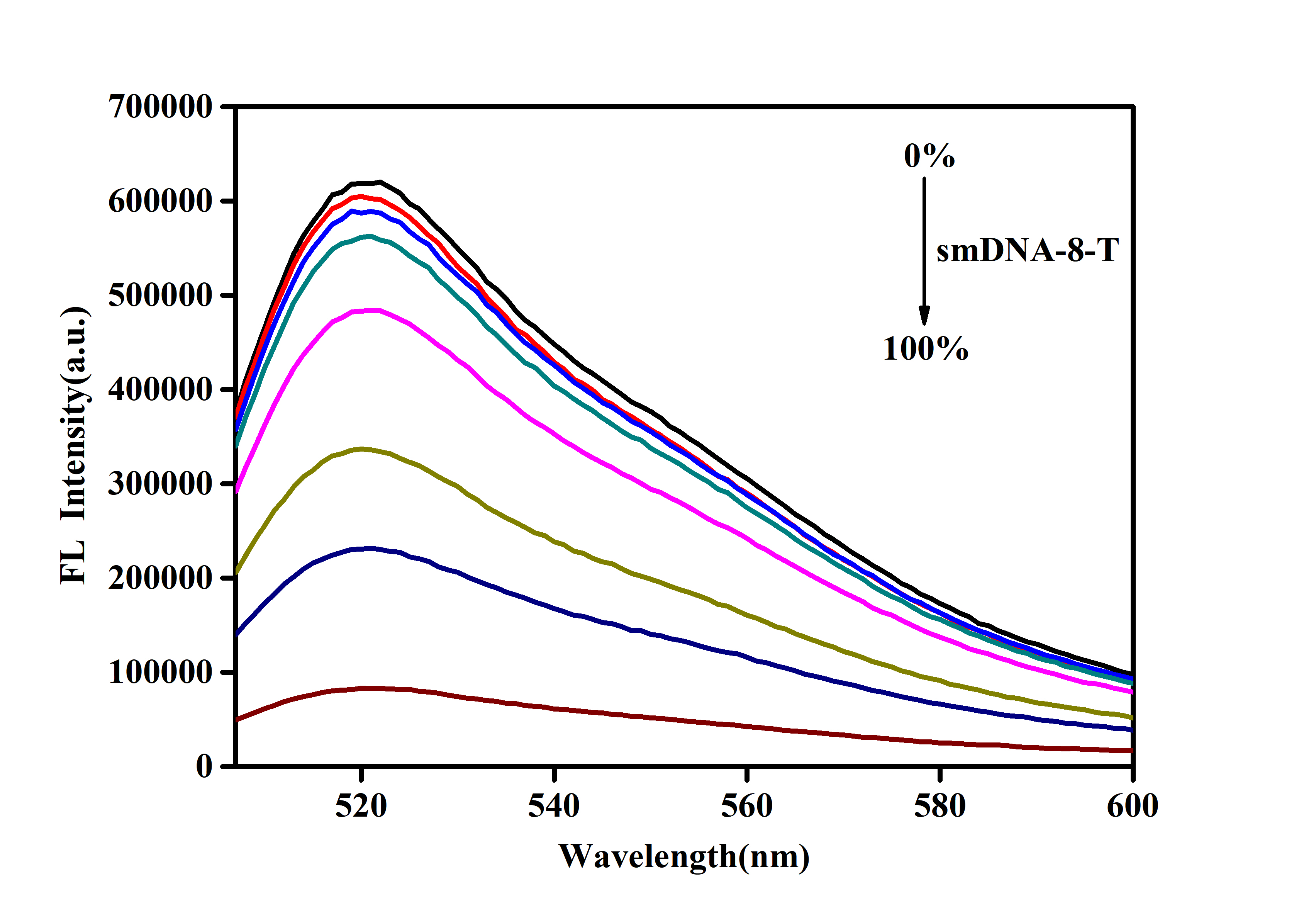

Supplement: Supplementary file 1 [file datasheet1.zip › Supplementary Figures/S6-2.jpg]

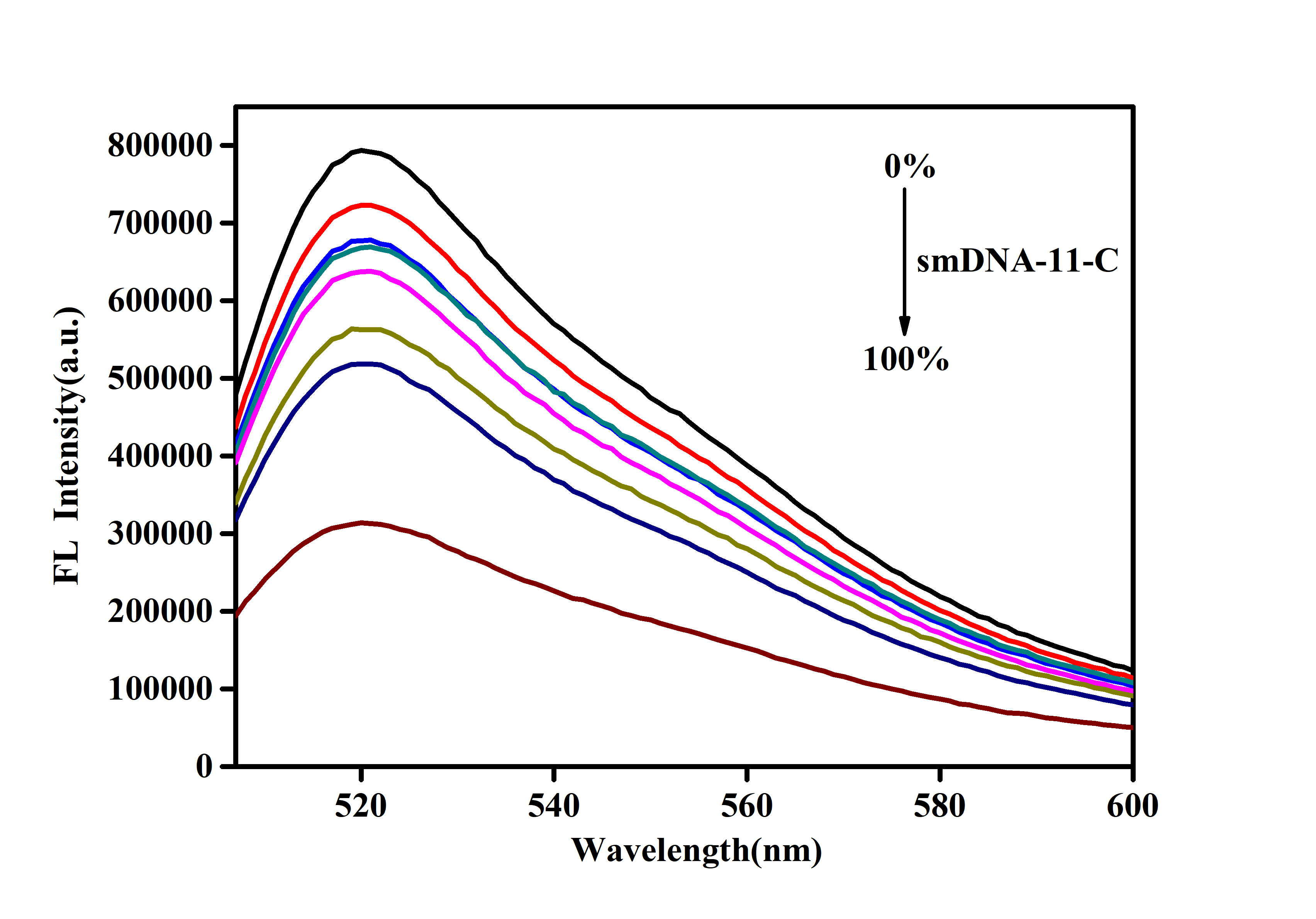

Supplement: Supplementary file 1 [file datasheet1.zip › Supplementary Figures/S6-3.jpg]
